# Selective Styrene Oxidation to Benzaldehyde over Recently Developed Heterogeneous Catalysts

**DOI:** 10.3390/molecules26061680

**Published:** 2021-03-17

**Authors:** Marta A. Andrade, Luísa M. D. R. S. Martins

**Affiliations:** Centro de Química Estrutural and Departamento de Engenharia Química, Instituto Superior Técnico, Universidade de Lisboa, 1049-001 Lisbn, Portugal; marta.andrade@tecnico.ulisboa.pt

**Keywords:** styrene oxidation, benzaldehyde selectivity, metal complexes, solid supports

## Abstract

The selective oxidation of styrene under heterogeneous catalyzed conditions delivers environmentally friendly paths for the production of benzaldehyde, an important intermediate for the synthesis of several products. The present review explores heterogeneous catalysts for styrene oxidation using a variety of metal catalysts over the last decade. The use of several classes of supports is discussed, including metal–organic frameworks, zeolites, carbon materials and silicas, among others. The studied catalytic systems propose as most used oxidants tert-butyl hydroperoxide, and hydrogen peroxide and mild reaction conditions. The reaction mechanism proceeds through the generation of an intermediate reactive metal–oxygen species by catalyst-oxidant interactions. Overall, most of the studies highlight the synergetic effects among the metal and support for the activity and selectivity enhancement.

## 1. Introduction

**Scheme 1:**
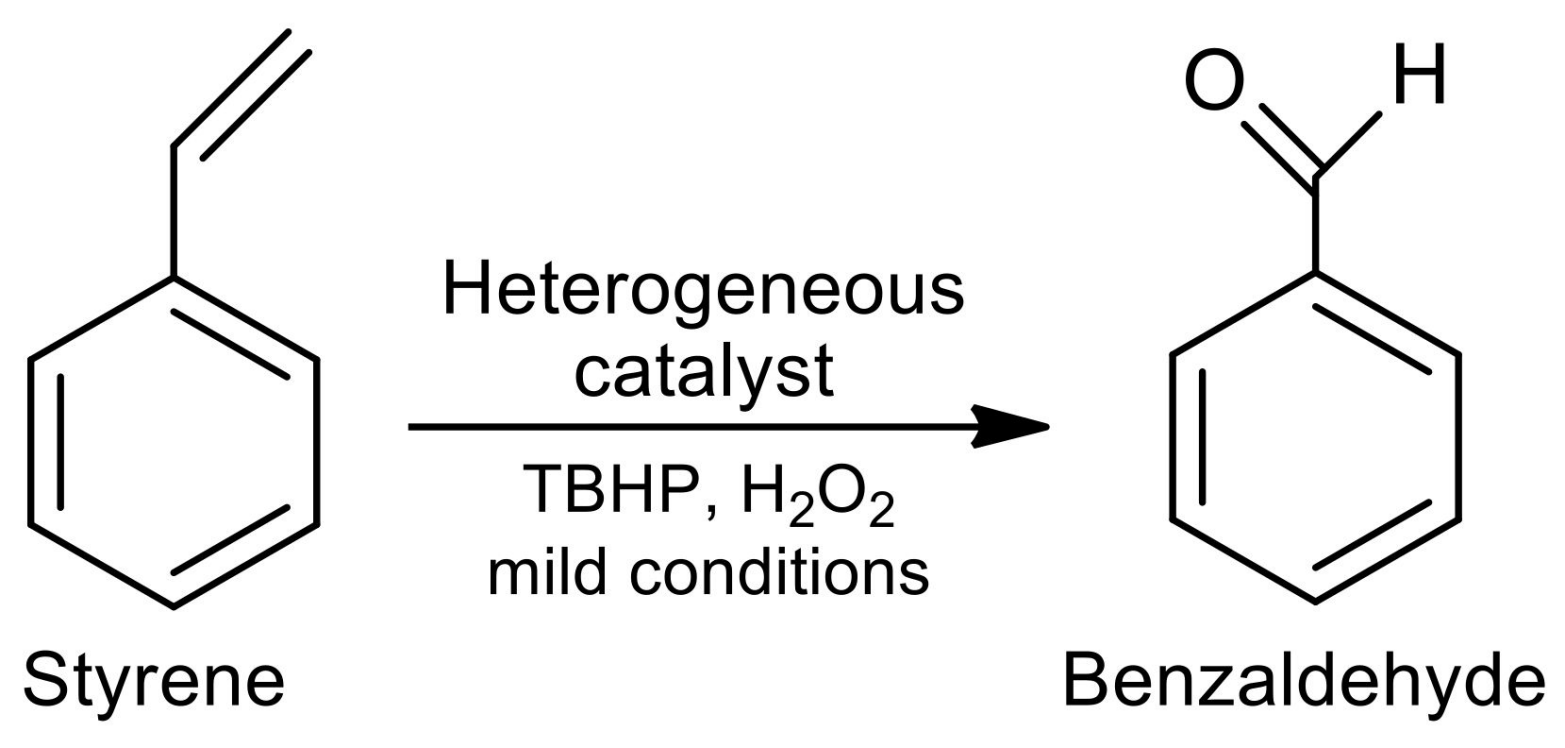
Styrene oxidation over heterogeneous catalysts under mild conditions.

Benzaldehyde is one of the most important and versatile organic compounds in the industry. This simple compound is widely used as an important intermediate for the synthesis of perfumes, epoxy resins, plasticizers, drugs, sweeteners and fine chemicals [1]. The industrial production of benzaldehyde is currently achieved by two processes, the catalytic oxidation of toluene to benzoic acid with oxygen and the hydrolysis of benzyl chloride [2,3]. However, both synthetic routes present important drawbacks (high temperatures and reaction times and/or production of chloride wastes), leading to low catalytic conversion and poor benzaldehyde yield (up to 20%) and selectivity. The selective oxidation of styrene is, therefore, considered a more appealing process as opposed to more complex methods, harsh conditions, and toxic waste disposal of the implemented procedures. The development of sustainable, effective catalytic systems for benzaldehyde production is hence a task of high significance in both academic and industrial fields. Owing to its simple reaction procedure and environmental-benign nature, the direct oxidation of styrene to benzaldehyde using environmentally friendly oxidants (Scheme 1) has, therefore, aroused much attention. For this matter, many kinds of homogeneous and heterogeneous catalysts have been investigated for the selective oxidation of styrene to benzaldehyde. The use of catalysts in the homogeneous phase, however, is associated with some disadvantages, such as high cost, difficult separation, and low recycling performance, limiting their further application in the catalytic process. Heterogeneous catalysts are in general considered to be more promising than their homogeneous counterparts due to their easy separation and recycling, although some of these can also pose important issues, such as high-cost, complex or time-consuming preparation procedures and low catalytic performance.

Nevertheless, a wide array of heterogeneous catalysts have been employed for styrene oxidation aiming for greener and more efficient methods [4]. A series of catalysts, from metal oxides, transition metal coordination complexes, polyoxometalates (POMs) and metal nanoparticles (NPs) supported on several solid materials, such as metal–organic frameworks (MOFs), zeolites, carbon materials, silicas, among others, have been investigated for the oxidation of styrene [5,6,7,8,9]. Commonly used oxidants in these systems are *tert*-butyl hydroperoxide (TBHP) and also hydrogen peroxide, air or oxygen. Still, most of the reported studies are focused on the selective styrene oxidation to styrene oxide [10], and comparatively fewer studies are focused on benzaldehyde as the main reaction product. The mechanism for this reaction, as proposed by most of the authors, involves the formation of a reactive metal–oxygen species intermediate by interactions between the catalyst and oxidant. This review accounts for relevant contributions in this field that propose efficient catalytic systems with reusable catalysts for the selective production of benzaldehyde.

## 2. MOF-Based Heterogeneous Catalysts

Metal–organic frameworks (MOFs) represent a class of hybrid porous materials, assembled with metal ions/clusters and organic linkers, which feature crystalline porous structures, displaying microporosity and, in some cases, mesoporosity [11,12,13]. The field of MOF chemistry continues to expand rapidly, owing to the seemingly limitless number of potential structures, but also due to the myriad of potential applications, as hydrogen storage, carbon dioxide sequestration, smart sensors, drug delivery, with a special focus on catalysis [14]. MOF-derived porous materials are very promising materials for catalytic applications owing to their unique attributes, such as large surface area, high porosity, and excellent tailoring ability [8]. This class of functional solid-state materials combines both the virtues of heterogeneous and homogeneous catalysis, such as high-efficiency and selectivity, recyclability, etc. One attractive approach to the construction of catalytic MOFs, therefore, is the incorporation of effective catalyst molecules in homogeneous solution environments. The highly porous structures with well-dispersed active sites facilitate the access of reactants, while their highly uniform pore shapes and sizes confer their size-selective catalytic properties, their stable character, ensuring recyclability. The research advances of MOF-based heterogeneous catalytic systems for the selective styrene oxidation to benzaldehyde are discussed below, highlighting the key factors that contribute to the enhanced catalytic performance of these systems.

**Figure 1:**
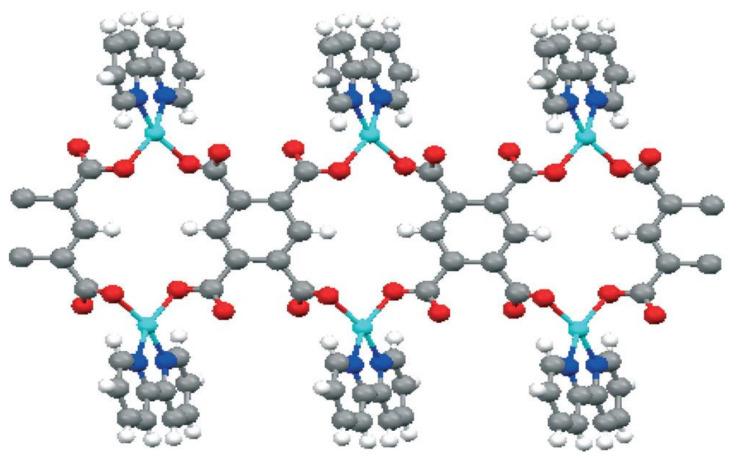
Structure of [Cu_2_(bipy)_2_(btec)]∞ catalyst. Reproduced with permission from [15]. Copyright (2015), The Royal Society of Chemistry, Amsterdam, The Netherlands.

In 2014, Cancino et al. reported the reusable Cu(II)-based MOF as a catalyst for the liquid phase oxidation of styrene [15]. The MOF [Cu_2_(bipy)_2_(btec)]_∞_ (bipy:2,2-bipyridin, btec:1,2,4,5-benzenetetracarboxylate), obtained by a hydrothermal technique (Figure 1) [16] was applied as a heterogeneous catalyst in the oxidation of styrene, with TBHP as the oxidant, either in water: dichloroethane or decane medium. The authors tested and compared two catalytic systems, dichloroethane–water and decane, after 6 h of reaction. A considerable activity for styrene oxidation was observed, achieving conversions between 61 and 45% for the system dichloroethane: TBHP: water system, and similar conversions when the solvent was replaced by decane, between 57 and 46%, under the same reaction conditions.

Although the system dichloroethane:TBHP: water [Cu_2_(bipy)_2_(btec)]_∞_was not selective for benzaldehyde when H_2_O_2_ was used, a 100% benzaldehyde selectivity was achieved (Table 1). The oxidation of styrene using [Cu_2_(bipy)_2_(btec)_∞_ has shown moderate to good conversion, as compared with some MOF and Cu(II) heterogeneous catalysts reported in the literature [15]. Negligible leaching was observed in the recycling studies, and the structure of the catalyst was preserved throughout four catalytic runs, as confirmed by the analysis of the spent catalyst by powder X-ray diffraction.

In this study, showing similar yields for oxidation products, the formation of benzaldehyde is achieved by a two-step mechanism that starts with the attack of (CH_3_)_3_OO^•^ on styrene, in accordance with a reported mechanism for the oxidation reaction using TBHP [17].

**Figure 2:**
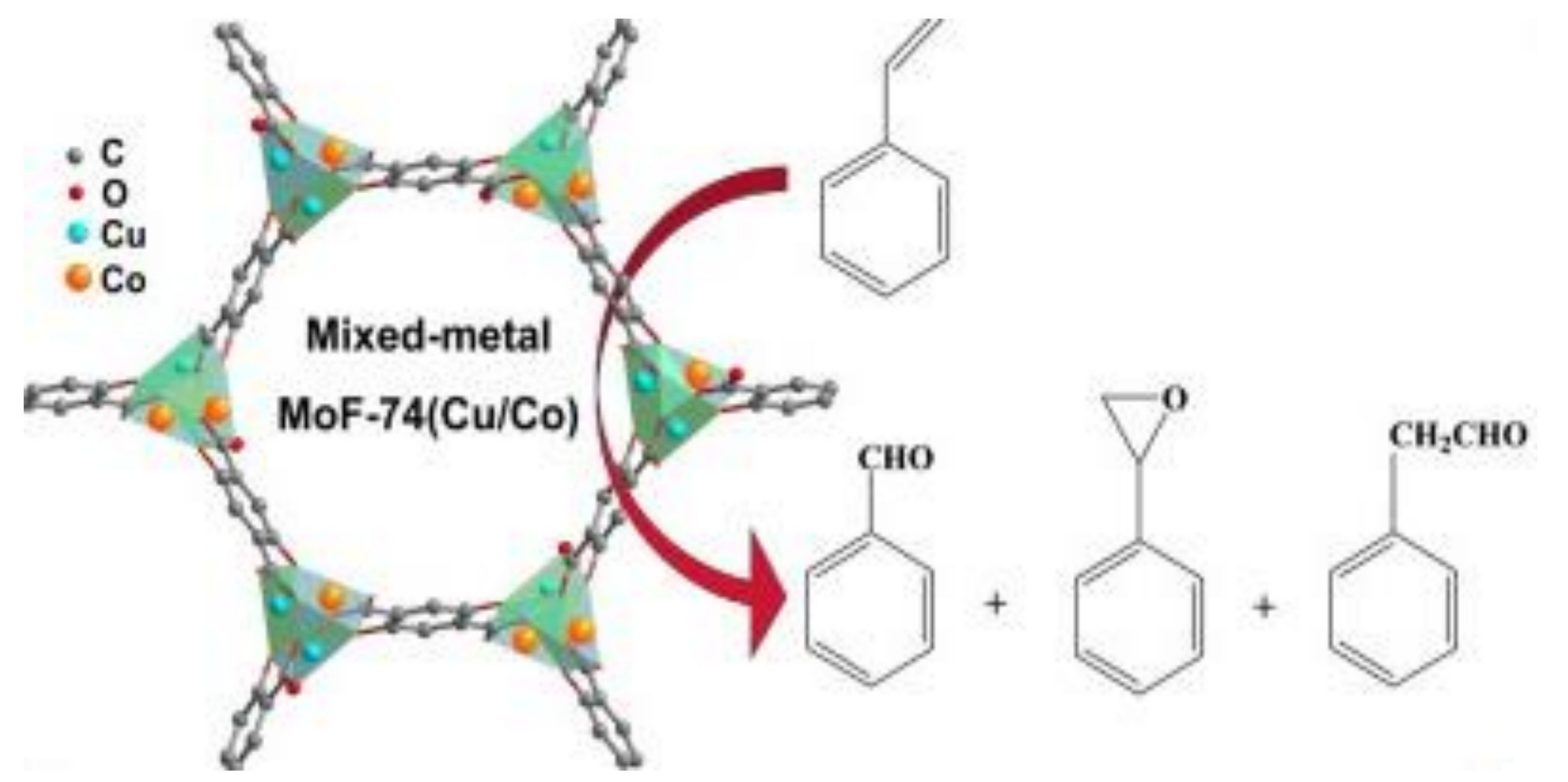
Cu and Co mixed metal–organic frameworks (MOFs) catalysts for styrene oxidation. Reproduced with permission from ref [18]. Copyright (2016), Elsevier B.V., Amsterdam, The Netherlands.

In 2016, Fu and coworkers explored the tunable catalytic properties of multi-MOFs for styrene oxidation, reporting a strategy for the incorporation of multiple metal cations into these materials [18]. The strategy of multivariate MOFs has drawn much scientific interest, given the possibility to introduce multiple functionalities into a single MOF network [19,20]. In this sense, Cu and Co mixed MOFs, MOF-74(Cu/Co), were prepared through a one-pot synthesis and applied to styrene oxidation under mild reaction conditions in the absence of solvent (Figure 2).

Regardless of the Cu/Co ratios, a uniform distribution of both Cu^2+^ and Co^2+^ was observed in MOF-74. The results showed that the catalytic properties could be tuned by varying the Cu/Co ratio in MOF-74 (Table 2). While for MOF-74(Cu), a relatively low catalytic activity, although accounting for an absolute selectivity for benzaldehyde was obtained, MOF-74(Co) demonstrated a rather higher catalytic activity, which led to the formation of benzaldehyde, styrene epoxide, and phenylacetaldehyde as well as to polystyrene. Upon incorporation of Cu^2+^ in MOF-74(Co), the polymerization of styrene was completely inhibited, whereas the incorporation of Co^2+^ in the structure of MOF-74(Cu) considerably enhanced the conversion to benzaldehyde, styrene epoxide, and phenylacetaldehyde.

In particular, the selectivity for benzaldehyde decreased from 75 to 43%, using MOF-74(Cu-90/Co-10) and MOF-74(Cu-30/Co-70), respectively. Moreover, synergetic effects of Co^2+^ and Cu^2+^ in MOF-74(Cu/Co) were demonstrated by the comparison of the catalytic properties of MOF-74(Cu/Co) with those of the physical mixtures of MOF-74(Cu) and MOF-74(Co). The reusability of MOF-74(Cu-30/Co-70) was studied, performing a six-cycle experiment, and only slight changes were observed for conversion and selectivity. Additionally, characterization of the spent catalyst attested for the stability of the prepared catalyst.

Ha and colleagues explored the catalytic performance of four MIL (MIL: Matériaux de l′Institut Lavoisier) MOF materials (Mn-MIL-100, Fe-MIL-100, Fe-MIL-101, Cr-MIL-101), that were obtained through a modified solvothermal methodology [21], presenting high surface area and stability as heterogeneous catalysts in the selective oxidation of styrene with TBHP in acetonitrile [22]. These previously reported mesoporous materials [21,23,24] exhibit high chemical and thermal stability as well as good resistance to air, water and common solvents. Despite some reports of their use in the oxidation of alkanes, their use in the heterogeneous oxidation of styrene was not explored to date. Solvent effect and operational parameters as temperature, catalyst dosage, reaction time and mole ratio were investigated. From the studied materials, Mn-MIL-100 exhibited excellent styrene conversion, reaching 89% with a benzaldehyde selectivity of 85% under optimized conditions (Table 3). Moreover, Mn-MIL-100 was reused for three consecutive runs without presenting substantial deactivation, demonstrating high chemical stability and recyclability. The high recyclability for the Mn-MIL-100 catalyst for selective styrene oxidation was proved through the reuse in three consecutive runs, with slightly reduced (<4%) styrene conversion and practically constant benzaldehyde yield.

A plausible mechanism for the selective oxidation of styrene over the studied catalysts was explored with the addition of a radical scavenger, butylhydroxytoluene, after 2 h of the catalyzed reaction with Mn-MIL-100. The immediate termination of the reaction upon BHT addition corroborated the participation of radical intermediates.

**Scheme 2:**
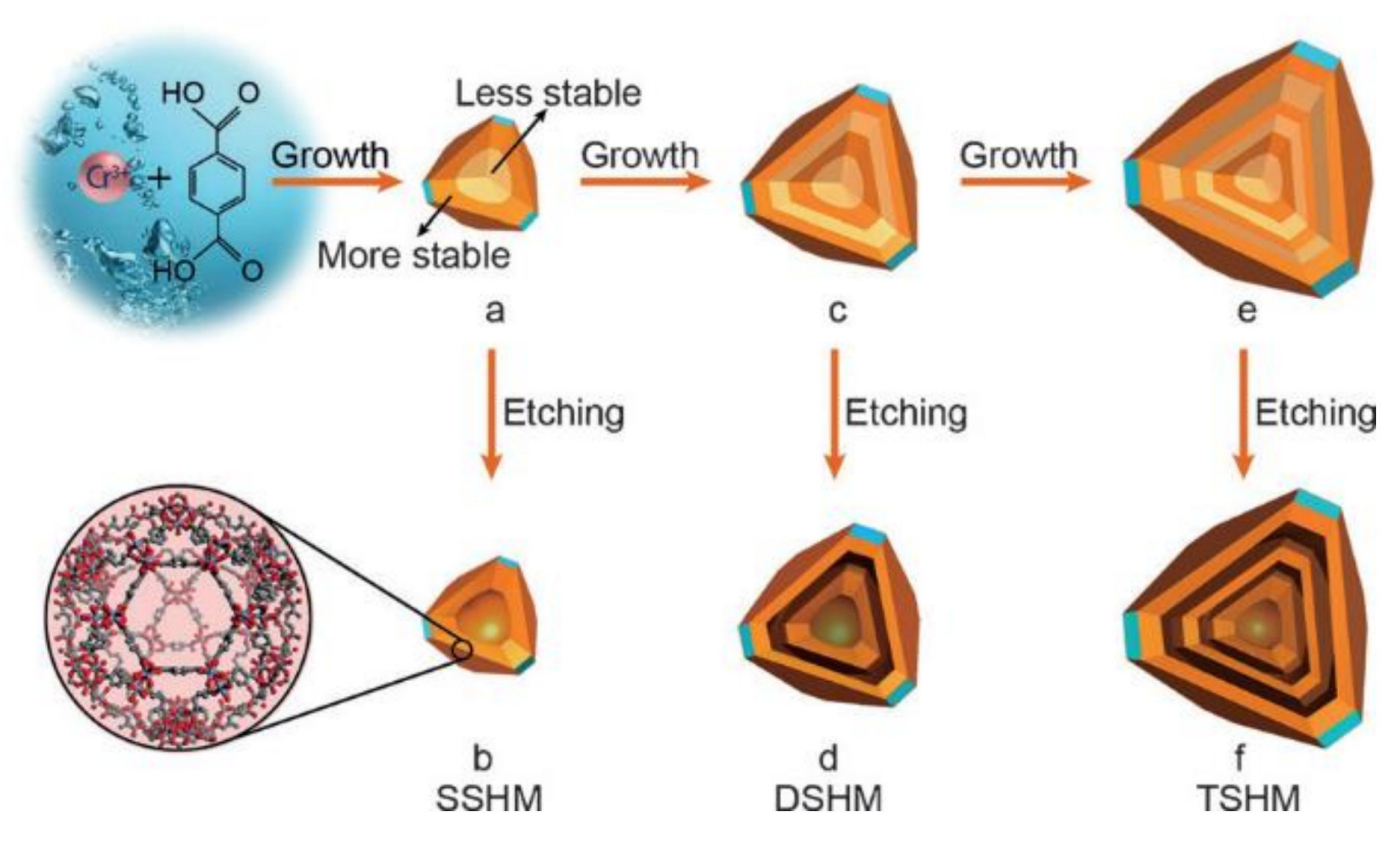
Fabrication of single-, double-, and triple-shelled hollow MIL-101, which are denoted as SSHM, DSHM and TSHM, respectively. Reproduced with permission from ref [25]. Copyright (2017), Wiley-VCH Verlag GmbH and Co. KGaA, Weinheim, Germany.

**Figure 3:**
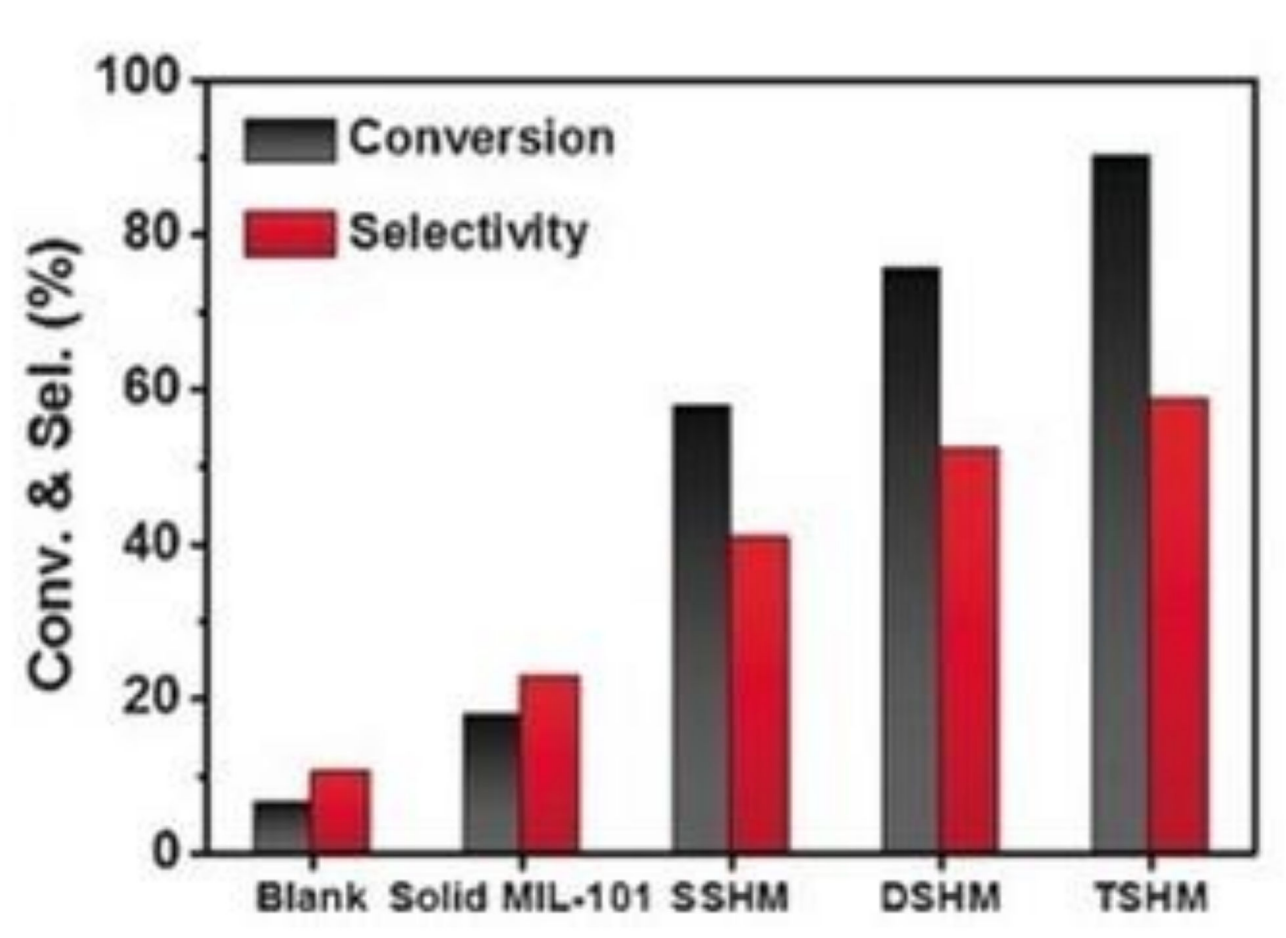
Styrene oxidation reaction catalyzed by different catalysts (Blank means no catalyst was used). Reproduced with permission from ref [25]. Copyright (2017), Wiley-VCH Verlag GmbH and Co. KGaA, Weinheim, Germany.

Liu et al. reported the first example of a catalytic system consisting of multi-shelled hollow MOFs (MSHMs) [25]. The hierarchically porous structures of MSHMs have received much attention and provided advanced applications in several fields [26]. The authors developed a method to fabricate hollow MIL-101 crystals with one, two or three shells, designated as SSHM, DSHM and TSHM, respectively, via step-by-step crystal growth and subsequent etching processes (Scheme 2). The accurate control of the size of the cavity and shell thickness was achieved by regulation of the etching time and the reactant concentration for each layer. Figure 3 illustrates the conversion and selectivity for the oxidation of styrene over solid MIL-101, SSHM, DSHM, and TSHM crystals. The hollow MIL-101 exhibited a much higher activity than the MIL-101 crystal, being related to the number of layers in the MSHMs. A 90% styrene conversion was obtained after 12 h for TSHM, while, in comparison, 18% of styrene conversion was obtained using solid MIL-101.

The conversion rate observed for TSHM was much higher than that of solid MIL-101, pointing out that the hollow multi-shelled structure could substantially enhance the catalytic performance. Additionally, the MSHMs displayed an enhanced benzaldehyde selectivity in comparison to solid MIL-101. The catalytic results for the oxidation of styrene highlighted the importance of the design of the MSHMs to the outstanding catalytic results when compared to that of the solid nanocrystals. The recyclability of TSHM was also investigated by the authors, which observed relation between conversion and selectivity to benzaldehyde as a function of the number of consecutive catalytic cycles (Figure 3). No significant changes in terms of the catalytic activity and selectivity after five successive cycles of reuse were reported.

**Scheme 3:**
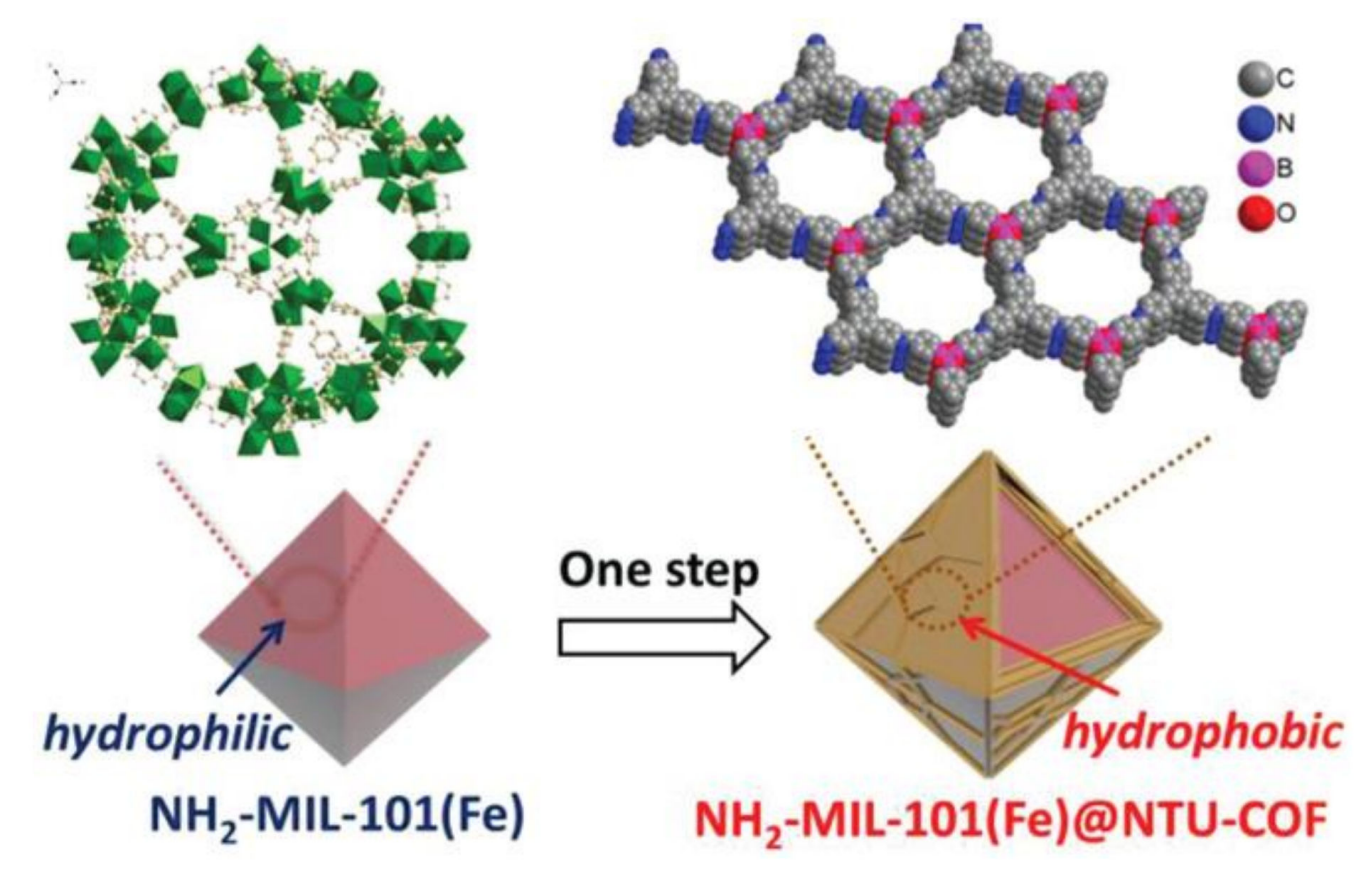
Fabrication of NH_2_-MIL-101(Fe)@NTU-COF. Reproduced with permission from ref [27]. Copyright (2019), Wiley-VCH Verlag GmbH and Co. KGaA, Weinheim, Germany.

Cai et al. prepared stable core–shell composites, consisting of a mesoporous MOF, NH_2_-MIL-101(Fe) as a core and a mesoporous covalent organic frameworks (COFs) shell denoted as MOFs@COFs for the oxidation of styrene [27]. The integration of MOFs and COFs in this new type of MOF@COF core–shell material, presenting high crystallinity as well as a hierarchical pore structure, was recently reported [28]. These materials were synthesized via a one-step modification, enabling hierarchical crystalline porous materials with adjustable hydrophilic–hydrophobic properties (Scheme 3). The composite NH_2_-MIL-101(Fe)@NTU obtained by a covalent linking process of NUT-COF-1 (NTU) (NTU: Nanyang Technological University) kept his retentive crystallinity with a hierarchical porosity and showed a considerably enhanced conversion and selectivity for styrene oxidation. The pore environment and the hydrophilic–hydrophobic properties of the hydrophobic NTU-COF shell were successfully modified, boosting the radical nature mechanism path after combining the hydrophilic MOF nanocrystals with the hydrophobic COFs shell.

**Figure 4:**
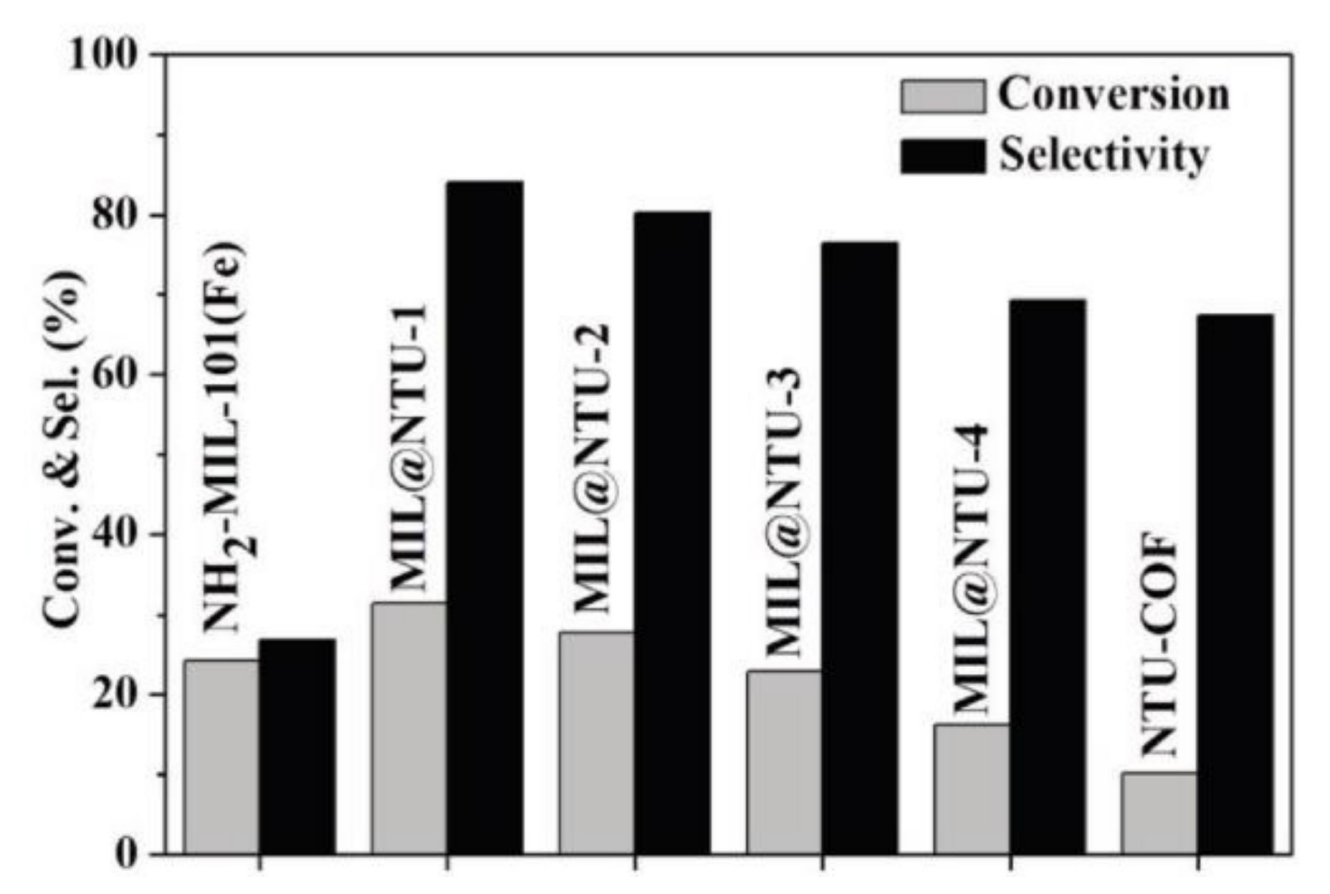
Styrene oxidation reaction catalyzed by different catalysts. Reproduced with permission from ref [27]. Copyright (2019), Wiley-VCH Verlag GmbH and Co. KGaA, Weinheim, Germany.

MIL@NTU-1 exhibited 84% of selectivity at 32% of styrene conversion, higher than those of 26% and 24% for the NH_2_-MIL-101(Fe), respectively (Figure 4). The enhanced catalytic performance was mainly ascribed to the coordinative unsaturated catalytic sites provided by NH_2_-MIL-101(Fe), while the NTU-COF shell promotes the radical mechanism for styrene directly to benzaldehyde, as it gathers the hydrophobic molecules substrate. Likewise, to verify the superiority of the core–shell structure, simple physical mixtures of NH_2_-MIL-101(Fe) and NTU-COF, containing the equal catalytic centers CUSs to MIL@NTU-1, were also tested in the same system. Lower conversion and selectivity were observed for the physical mixture compared to the core–shell structured MIL@NTU-1, pointing out the significance of the structure for MIL@NTU in the transfer process.

This high selectivity was imputed to the synergistic effect between the MOF core and COF shell. To address the catalytic stability of MIL@NTU-1, recycling experiments were performed for four successive cycles. No obvious changes in terms of the catalytic activity and selectivity were observed for the catalyst, maintaining its octahedral morphology and rough surface.

**Figure 5:**
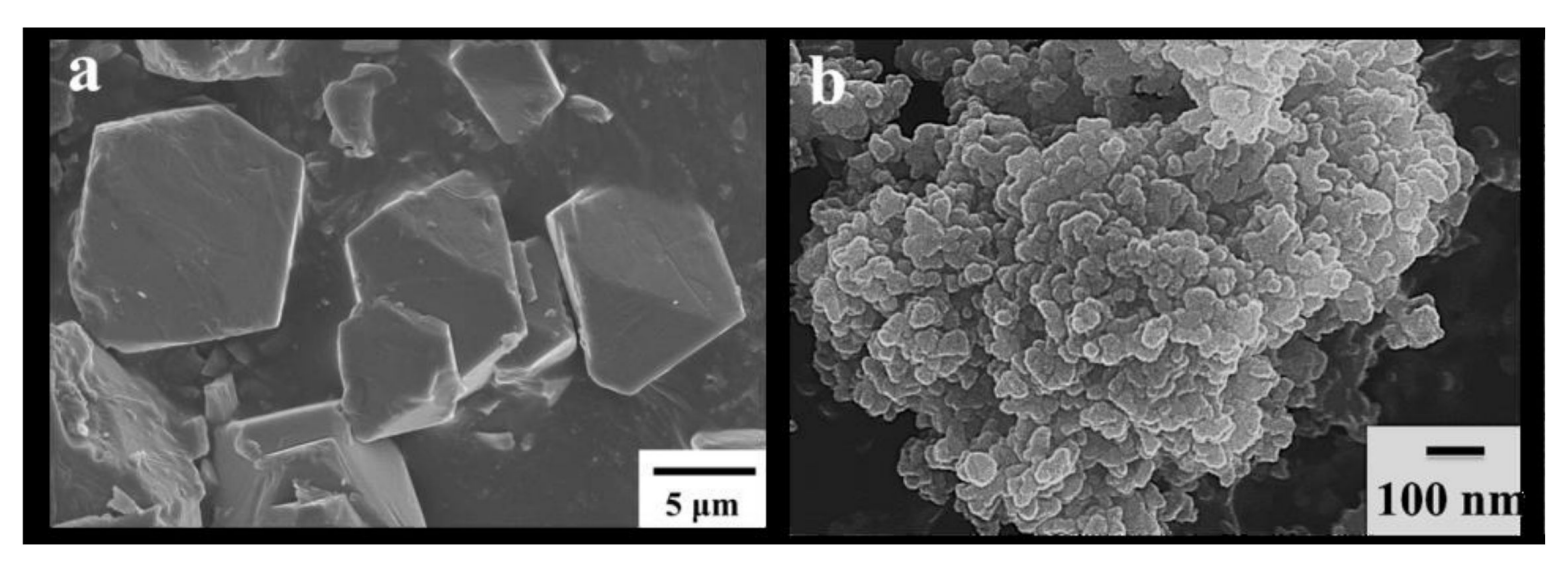
(**a**) SEM of the bulk HKUST-1; (**b**): SEM of the nano HKUST-1. Reproduced with permission from ref [27]. Copyright (2018), American Chemical Society, Amsterdam, The Netherlands.

Guo and coworkers explored the use of HKUST-1 (HKUST: Hong Kong University of Science and Technology), a well-established MOF in the field of hydrogen gas storage and heterogeneous catalysis owing to its textural parameters and high thermal stability [29]. The authors reported the 2-methylimidazole-assisted synthesis of nanosized HKUST-1, Cu_3_(BTC)_2_ (BTC: 1,3,5-benzene tricarboxylate [30]. The addition of 2-MI resulted in nanosized HKUST-1, presenting a size range of 10–20 nm (Figure 5). The presence of 2-MI tuned its morphology and improved its catalytic activity.

**Scheme 4:**
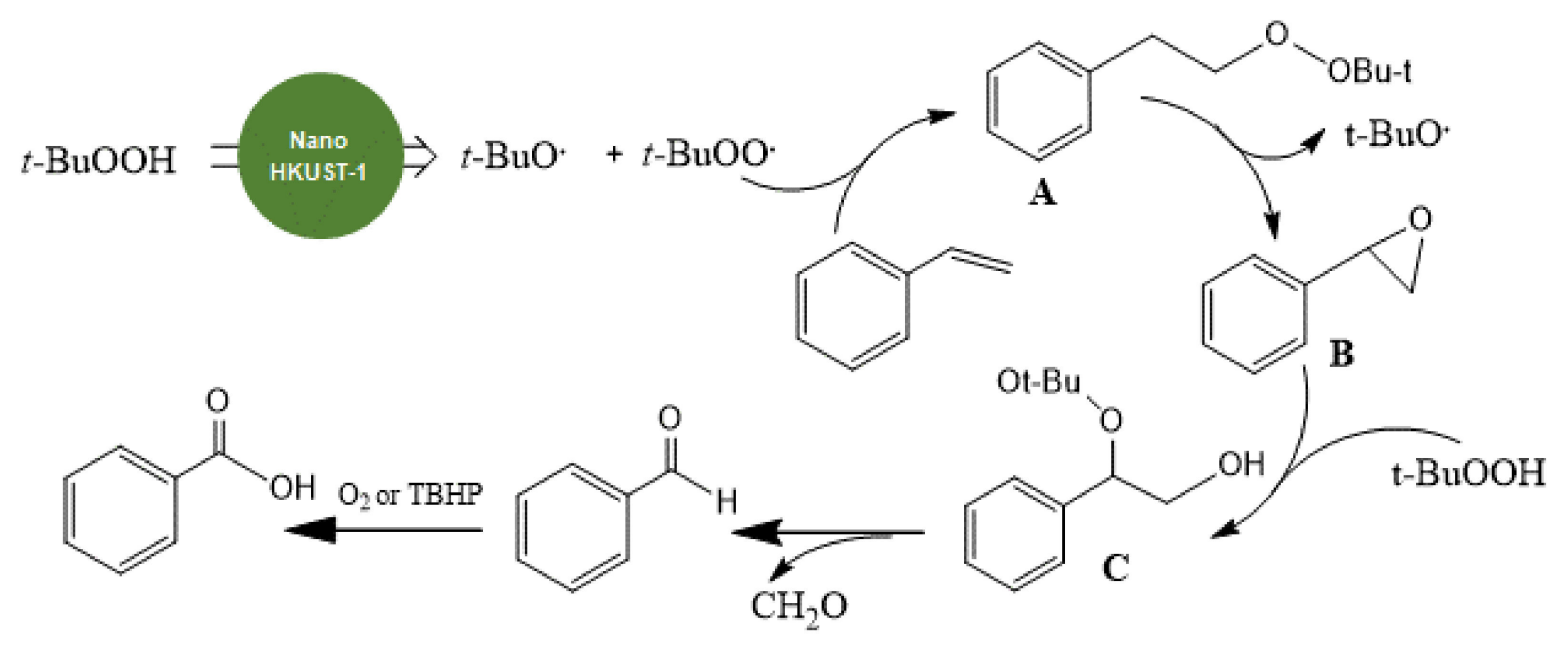
Proposed mechanism for styrene oxidation over nano HKUST-1 [30].

Catalytic experiments demonstrated the higher catalytic activity of nanosized HKUST-1 when compared with bulk HKUST-1 towards solvent-free styrene oxidation using TBHP. A simple variation of the reaction parameters allowed the successful production of benzaldehyde, with 78% yield after 9 h at 50 °C or 72% after 50 min at 80 °C. Furthermore, gram scale experiments were also performed, revealing the general applicability of this oxidation procedure. For this matter, 30 mg of the catalyst and 15 mmol of TBHP were sufficient in this gram-scale reaction. The nano HKUST-1 was conveniently recycled four times without almost unaffected benzaldehyde yield. According to the obtained results and previously reported literature reports [31,32], the authors proposed a possible reaction pathway (Scheme 4). The mechanism involves the generation of tert-butoxy and TBHP radicals by TBHP decomposition with nano HKUST-1 catalyzed single electron transfer processes and further reaction with styrene affording intermediate A, that upon release of the *t*-BuOH generates styrene epoxide. Finally, the decomposition of intermediate C, 2-(tert-butylperoxy)-2-phenylethan-1-ol (obtained by reaction of styrene epoxide with TBHP), yields benzaldehyde.

Ma et al. explored the dispersion of POMs, materials that have shown good catalytic performance for the selective oxidation of alkenes [33], into MOFs, increasing its low specific surface area and enabling its recyclability, thus broadening its application as heterogeneous catalysts [34]. The authors reported the synthesis of two POMOFs, [Co(BBTZ)_2_] [H_3_BW_12_O_40_]·10H_2_O and [Co_3_(H_2_O)_6_(BBTZ)_4_][BW_12_O_40_]·NO_3_·4H_2_O, (BBTZ = 1,4-bis(1,2,4-triazol-1-ylmethyl)benzene), denoted as POMOF1 and POMOF2, respectively, for the production of benzaldehyde.

**Figure 6:**
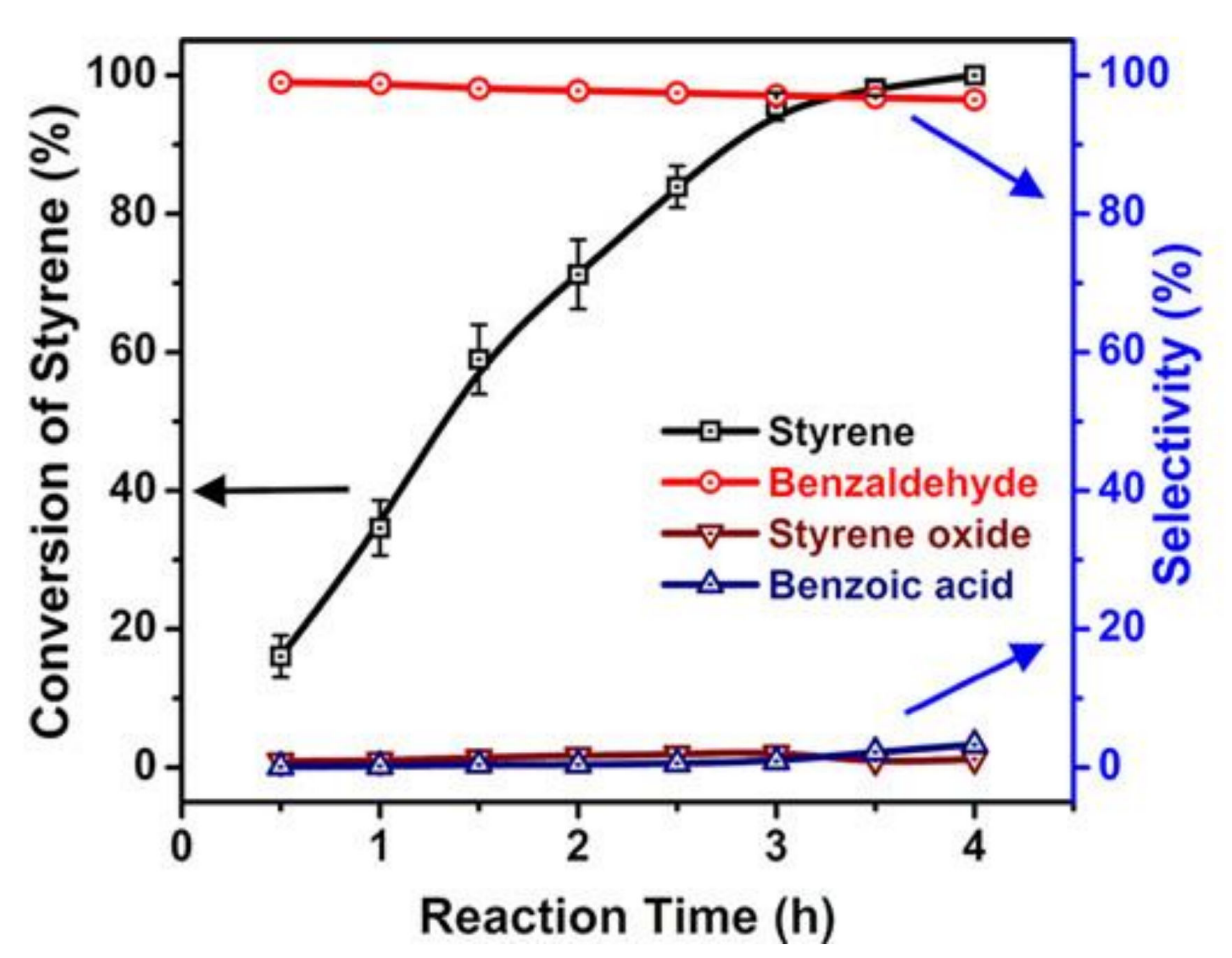
Conversion of styrene and selectivity of products in the selective oxidation of styrene with POMOF1 as a catalyst. (Reaction conditions: styrene (0.25 mmol), H_2_O_2_ (1 mmol), catalysts (0.028 mmol), and acetonitrile (2 mL) at 70 °C. Reproduced with permission from ref [34]. Copyright (2018), American Chemical Society, Amsterdam, The Netherlands.

The prepared catalysts achieved a 100% styrene conversion with 96% selectivity towards benzaldehyde (Figure 6). The co-role of [BW_12_O_40_] ^5−^ and Co^2+^ active center and a more open framework feature copromote the catalytic properties of the POMOFs. The recyclability of the materials as heterogeneous catalysts was also investigated, being reused in seven cycles without significant loss of activity, demonstrating good stability and maintaining its structural integrity. The authors postulated that the large porosity of POMOFs and their catalytically active POM units, as well as extended organic bridging ligands, could produce more high-performance catalysts in the oxidation reaction of alkenes to aldehydes.

**Scheme 5:**
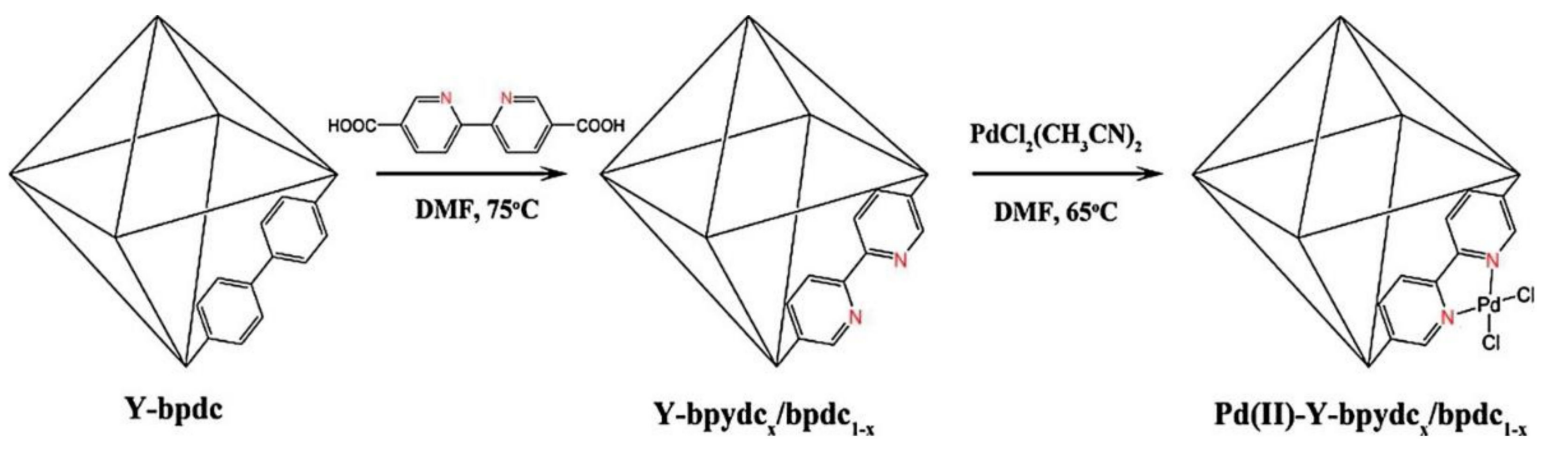
Synthesis of Pd(II)-Y-bpydc_x_/bpdc_1−x_ via sequential postsynthetic ligand exchange and metalation process. Reproduced with permission from ref [37]. Copyright (2020), Elsevier B.V., Amsterdam, The Netherlands.

Zhang et al. prepared a series of PdCl_2_ moiety-decorated Y_6_-MOFs using a post-synthetic strategy [35,36] for application as single-site catalysts for the selective oxidation of styrene [37]. In particular, the incorporation of the functional organic linker, H_2_bpydc (2,2′-bipyridine-5,5′-dicarboxylic acid), into Y(III)_6_ clusters-supported MOF, [(CH_3_)_2_NH_2_]_2_ [Y_6_(*μ*_3_-OH)_8_(bpdc)_6_] (bpdc = 4,4′-biphenyl dicarboxylic acid) via a postsynthetic ligand exchange, followed by Pd(II) introduction to enhance the density of single sites (Scheme 5).

The catalytic performance of Y_6_-MOFs presenting different Pd(II) loads, Pd(II)-Y-bpydc_x_/bpdc_1−x_, was investigate in the solvent-free styrene oxidation under mild conditions using O_2_ as oxidant (Table 4). Although a significant increase in styrene conversion was observed with the temperature increase (from 25 to 120 °C), the selectivity reached an inflection point at 80 °C, achieving its maximum. The introduction of Pd(II) significantly improved the catalytic activity of the Y_6_-MOF, as Y-bpdc and Y-bpydc_0_._8_/bpdc_0_._2_ showed no catalytic activity, confirming the important role of the immobilized Pd(bpydc)Cl_2_ species. PdCl_2_ (0.024 mmol) was also tested as a homogeneous catalyst for the oxidation of styrene, obtaining the lower conversion of styrene and benzaldehyde yield (54 and 78%, respectively). In this case, the conversion reached ca. 90% after 7 h, although a reduction of benzaldehyde selectivity occurred (65%).

Pd(II)-Y-bpydc_0_._8_/bpdc_0_._2_ catalyst exhibited higher catalytic activity, achieving styrene conversion and selectivity for benzaldehyde of 89 and 82%, respectively, after 5 h, outperforming some MOF-based catalysts. These results compare favorably, under similar reaction conditions to the selectivity (35%) achieved by MOF-74(Co), discussed above [15]. MIL-MOFs showed an inferior catalytic activity for the oxidation of styrene, that although presented a selectivity to benzaldehyde over 90%, achieved only 47% of conversion [22]. TSHM crystals also provided high styrene conversion (ca. 90%) but require longer reaction times (12 h) and TBHP as oxidant, with benzaldehyde selectivity of just ca. 60% [18], lower than that for Pd(II)-loading Y-bpydc_x_/bpdc_1−x_. A styrene conversion of 62 and 45% were observed for Pd(II)-Y-bpydc_0_._5_/bpdc_0_._5_ (0.015 mmol Pd) and Pd(II)-Y-bpydc_0_._2_/bpdc_0_._8_ (0.006 mmol Pd), with a high selectivity to benzaldehyde (86 and 90%, respectively). The decreasing amount of Pd(II) sites in the framework led to a marked decline in the conversion of styrene, nevertheless, with an increasing trend for selectivity to benzaldehyde. Pd(II)-Y-bpydc_x_/bpdc_1−x_ MOFs posed as good candidates for efficient catalysts for the selective oxidation of styrene through modulation of the incorporated Pd(II) load into the framework of these materials.

## 3. Zeolite-Based Heterogeneous Catalysts

Zeolites are crystalline materials that are widely used in heterogeneous catalysis, among other fields, as adsorption and separation processes [4,38,39]. The uniqueness of these materials relies on features as high porosity, mechanical stability, inherent acidity, shape selectivity, and exchange capacity, making them attractive candidates as hosts for encapsulation of metal complexes. Nevertheless, their intrinsic microporosity character presents diffusion limitations, the reason why their widespread use as catalytic supports is somewhat limited in comparison with other mesoporous materials. To hamper this disadvantage, some approaches aiming for mass transfer enhancement have been attempted [40,41]. The development of hierarchical zeolites via post-synthesis treatments that result in microporosity enlargement and mesoporosity generation efficiently combines the properties of the intrinsic zeolite with mesoporosity. Some recent applications of zeolites in catalytic systems for the oxidation of styrene are discussed.

**Figure 7:**
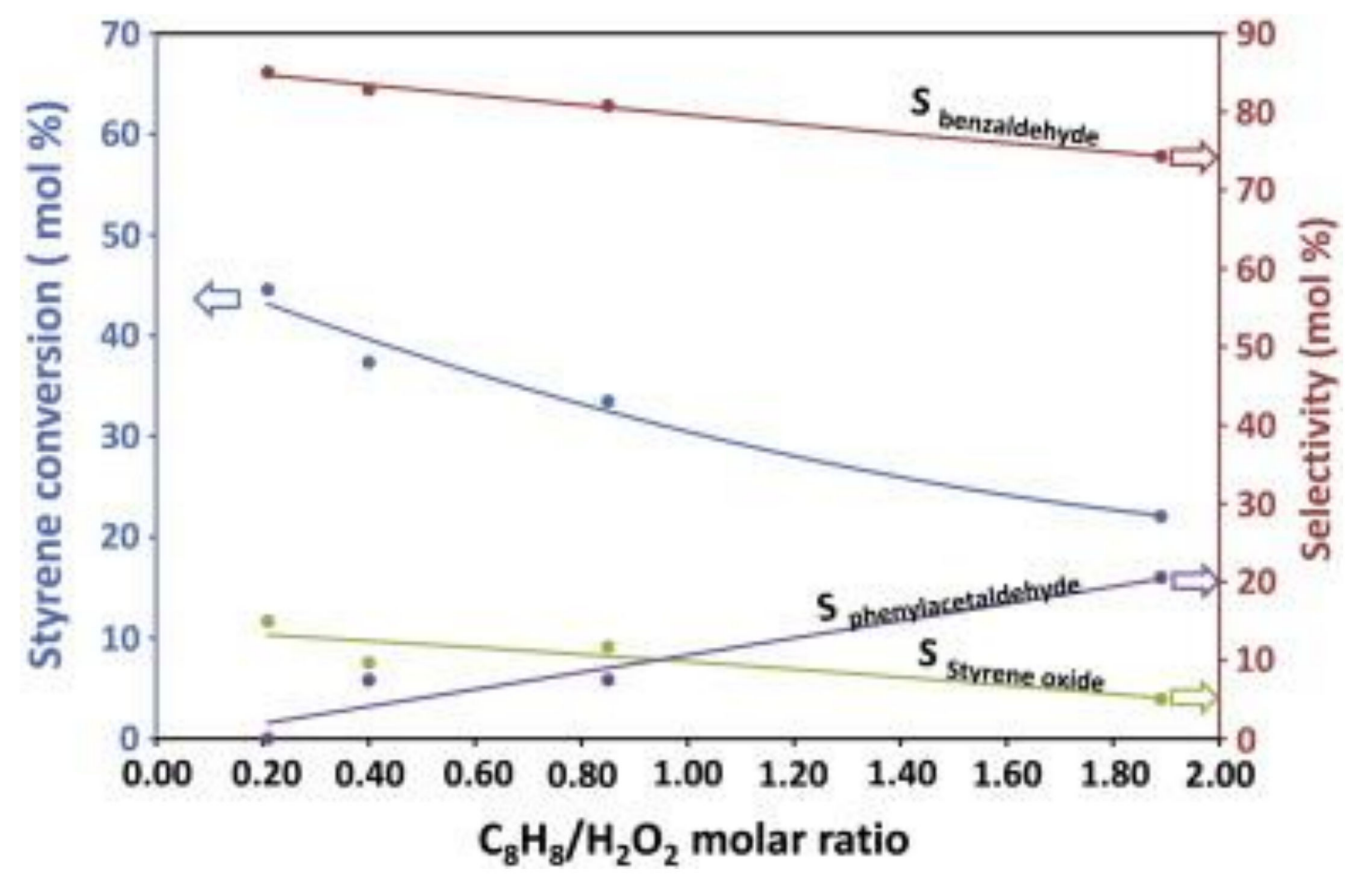
Effect of styrene (C_8_H_8_)/H_2_O_2_ molar ration on styrene conversion and selectivity to benzaldehyde, styrene oxide and phenylacetaldehyde. Reaction conditions: 0.4 g of Co-ZSM-11, T = 60 °C; 350 rpm. Reproduced with permission from ref. [42], Copyright (2013), Elsevier B.V., Amsterdam, The Netherlands.

In 2014, Jury et al. reported the synthesis of a new and efficient Co-ZSM-11 catalyst for the selective styrene oxidation to benzaldehyde [42]. The ZSM-11 zeolite was synthesized by a hydrothermal process and further doped by the ionic exchange as previously reported [5], obtaining Co-ZSM-11 zeolite that was fully characterized. The styrene oxidation catalytic studies were carried out using H_2_O_2_ as an oxidizing agent in acetonitrile, under microwave irradiation, in a system with a suspension of fine particles of Co-ZSM-11. The authors performed the optimization of several reaction parameters. An increase in styrene conversion and reaction rate was observed with increasing catalyst mass. The initial reaction rate was not proportional to the catalyst load due to the decomposition of hydrogen peroxide favored by an increase in the concentration of the catalyst. The optimal molar ratio substrate/oxidant was found to be 0.2 (Figure 7), with an optimal temperature of 60 °C. The Co-ZSM-11 catalyst presented a reaction rate about 30% higher when compared to those found in the literature and also a higher benzaldehyde selectivity of around 80% under optimal reaction conditions.

**Figure 8:**
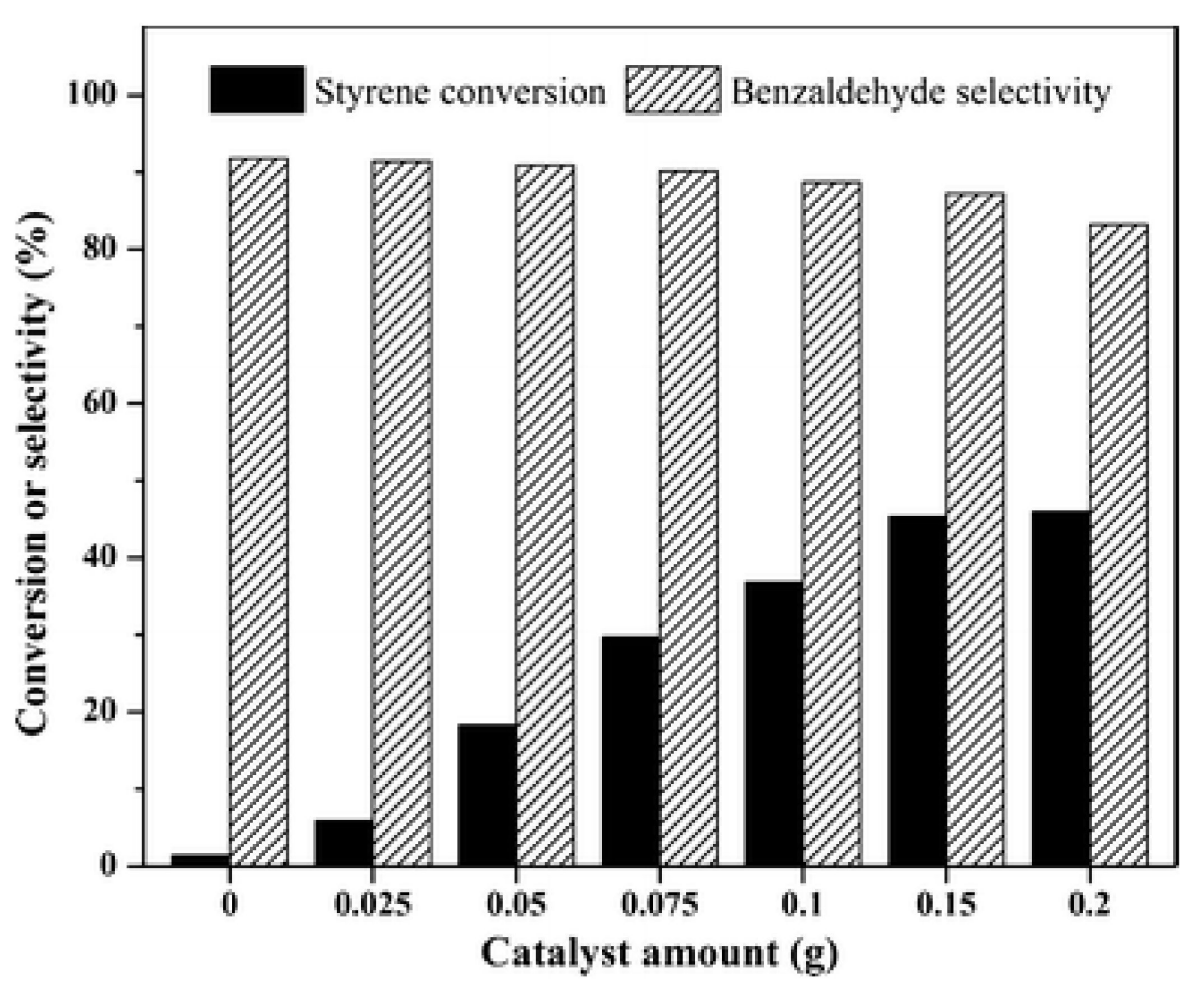
Influence of the catalyst amount on the oxidation of styrene (conditions: styrene 20 mmol, ZSM-5(60) catalyst, TBHP 30 mmol, acetonitrile 10 mL, 65 °C, time 6 h). Reproduced with permission from ref [43]. Copyright (2016) Springer, Amsterdam, The Netherlands.

In 2016, the catalytic performance of ZSM-5 catalysts using TBHP in the selective oxidation of styrene to benzaldehyde was evaluated by Narayanan and colleagues [43]. Hierarchical ZSM-5 catalysts presenting distinct Si/Al ratios (20, 60 and 100) were synthesized through a hydrothermal method and further characterized, confirming the crystallinity and average crystallite size decrease with an increasing Si/Al ratio [44]. Benzaldehyde was obtained as the major oxidation product, under optimum reaction conditions, given the rapid conversion of styrene oxide (Figure 8).

High selectivity to benzaldehyde was obtained by optimization of the reaction parameters, as the effect of Si/Al ratio, oxidant (TBHP, H_2_O_2_ and NaOCl), catalyst load, temperature and TBHP/styrene ratio. Among the catalysts, the ZSM-5(60) catalyst showed outstanding catalytic activities in the selective oxidation of styrene when using TBHP as the oxidant and acetonitrile as a solvent, at 65 °C in 6 h. ZSM-5(60) catalyst was recovered and reused three times without significant activity and selectivity loss. The authors proposed a plausible mechanism for the catalytic reaction in that Lewis acid sites in ZSM-5 zeolite act as the active sites of the catalyst. In the first stage, the Al framework activates the highly unstable perohydroxyl anion (•OOH^–^), forming an Al peroxo complex. Styrene is attracted by Al, forming an intermediate that leads to a nucleophilic attack of the peroxo oxygen to the electron-deficient styrene, forming a highly unstable complex, that upon decomposition, generates epoxide (styrene oxide), that is converted into benzaldehyde. Therefore, the molar ratio of TBHP/styrene strongly influences benzaldehyde rate formation.

In 2016, Desai and coworkers developed studies regarding the catalytic styrene oxidation over zeolite-Y nanohybrid materials [45]. The authors prepared VO(IV), Mn(II), Fe(II), Co(II), Ni(II), Cu(II) metal complexes and a Schiff base ligand, HNIMMPP (4-(((2-hydroxy-5-nitrophenyl)imino)methyl)-3-methyl-1-phenyl-1H-pyrazol-5-ol), within zeolite-Y structure. Benzaldehyde is the major product obtained for the oxidation reaction of styrene with TBHP, with styrene glycol, chalcone and 2-phenyloxirane as minor products. The catalyst [VO(HNIMMPP)(H_2_O)]-Y provided the highest styrene conversion, 93% and selectivity to benzaldehyde of 46% (Table 5).

Negligible styrene conversion was reported in the presence of Na-Y zeolite, and metal exchanged zeolite-Y, suggesting the key role of the complexes in the oxidative reaction of styrene. The recyclability of the materials revealed that the entrapped complexes performed as heterogeneous catalysts. The catalytic reaction scheme proposed by the authors for the oxidative reaction of styrene over zeolite-Y imprisoned VO(IV) complexes could undergo two possible paths [46]. The attack of metal-oxo species to the C=C double bond of styrene furnishes an intermediate by oxidative addition of metal-oxo species ^•^OOV^5+^L/NaY, cyclic styrene oxide and regenerated OV^4+^L/NaY. Styrene oxide reacts with metal-oxo species to produce benzaldehyde.

**Figure 9:**
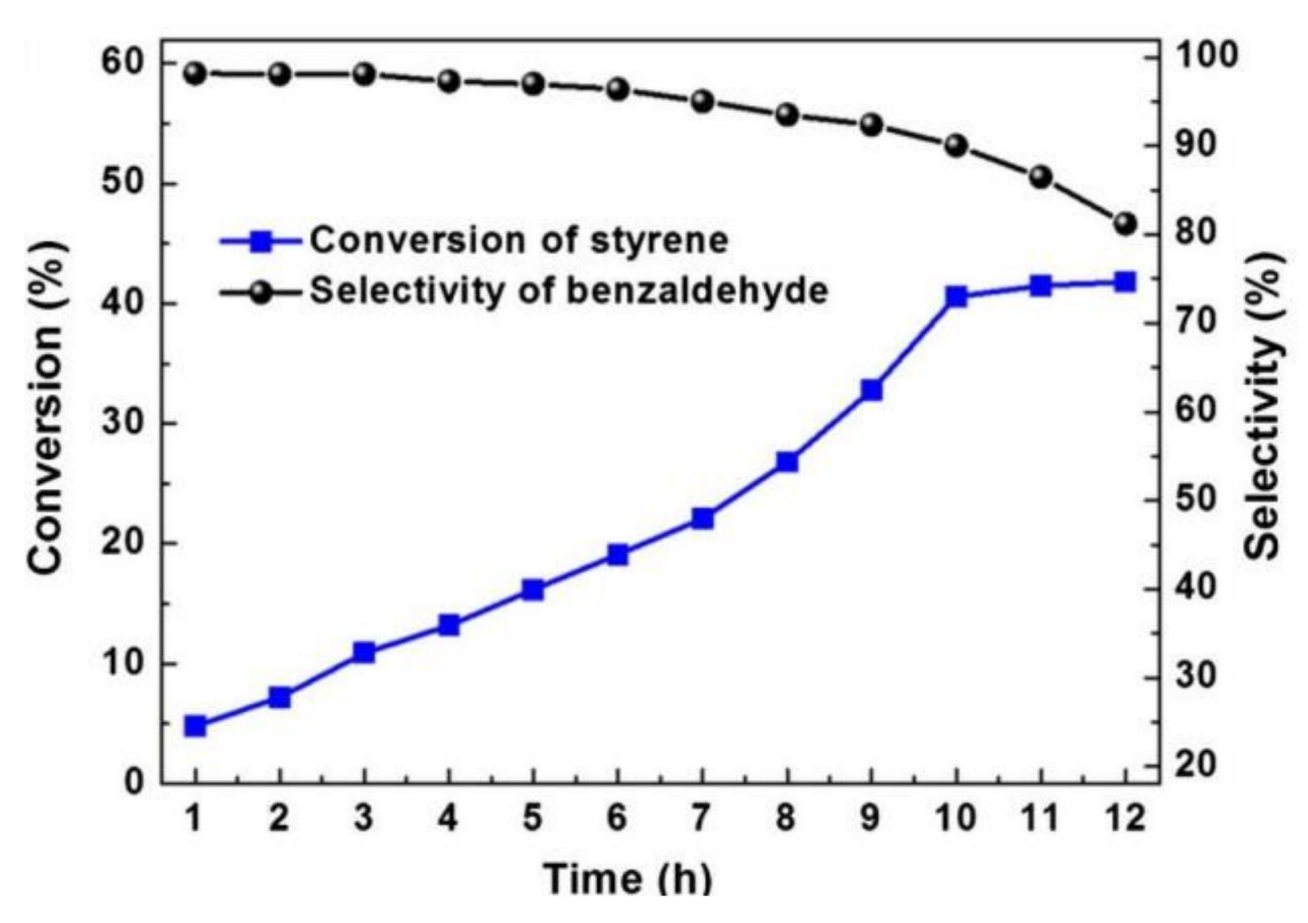
The effect of reaction time on the selective oxidation of styrene to benzaldehyde on styrene conversion and benzaldehyde selectivity. Reproduced with permission from ref [47]. Copyright (2018) Elsevier Inc., Amsterdam, The Netherlands.

A catalyst with encapsulated cobalt oxide particles into ZSM-5 zeolite (Co_3_O_4_@HZSM-5) was reported by Liu et al. [47]. The catalyst was hydrothermally obtained, with the impregnated Co_3_O_4_/SiO_2_ catalyst as the precursor and Si source. The Co_3_O_4_@HZSM-5 catalyst presented a well-organized structure with encapsulated Co_3_O_4_ particles within the zeolite crystals. The prepared material was used as a catalyst for the oxidation of styrene to benzaldehyde, using H_2_O_2_ (Figure 9).

The authors investigated the effect of several reaction parameters, including reaction time, temperature, solvent, styrene/H_2_O_2_ ratio and catalyst load on the catalytic performance. As expected, a much higher styrene conversion and benzaldehyde yield were observed for the Co_3_O_4_@-HZSM-5 catalyst compared to the precursor Co_3_O_4_/SiO_2_. Under the optimized reaction conditions, benzaldehyde yield achieved 79% with 97% styrene conversion and 82% of selectivity. This excellent catalytic activity was attributed to the synergy achieved between the unique Co_3_O_4_ encapsulated zeolite structure with a confined reaction environment and the acidic property since Co_3_O_4_/SiO_2_ and Co_3_O_4_@HZSM-5 present comparable surface area and Co_3_O_4_ particle size.

**Scheme 6:**
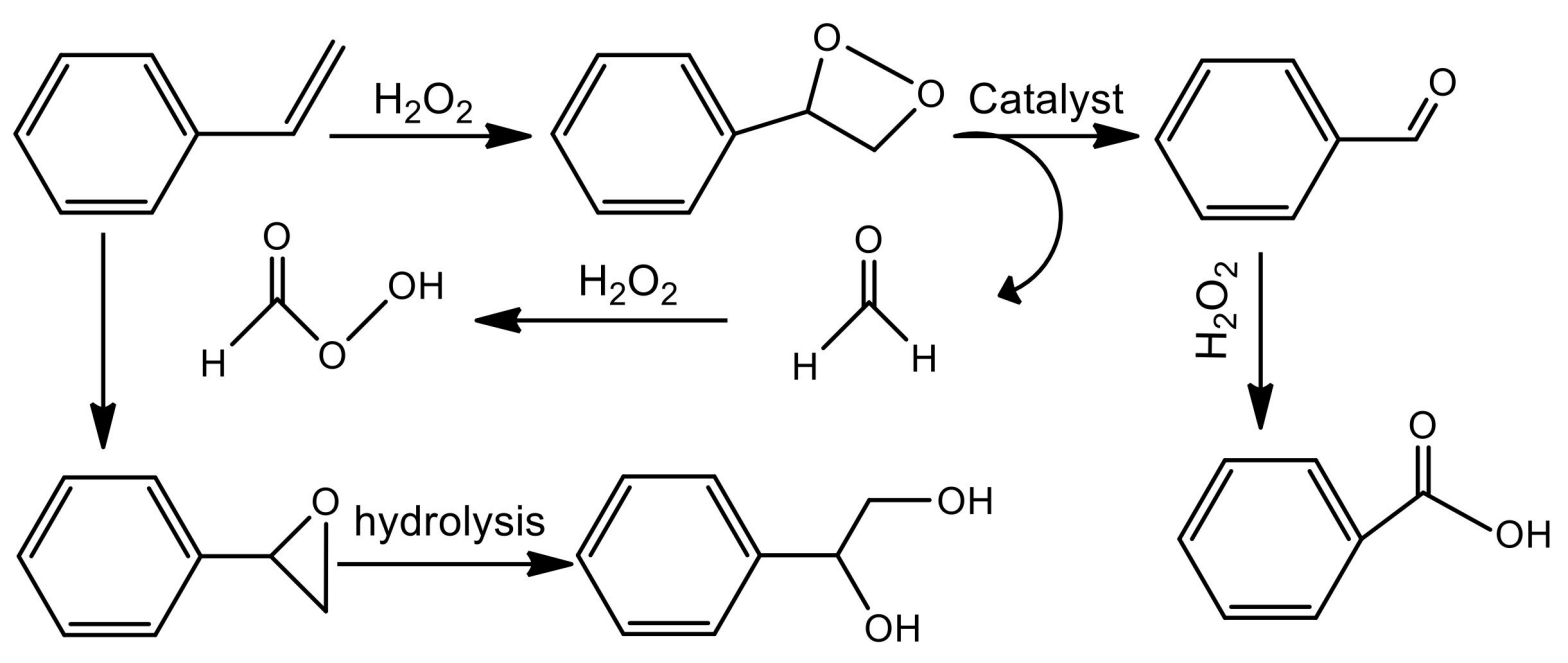
Proposed mechanism of styrene oxidation to benzaldehyde [47].

The pristine HZSM-5 zeolite as catalyst showed a very small reactivity, highlighting the importance of the introduction of cobalt oxide particles in the zeolite structure. The reaction mechanism with Co_3_O_4_@HZSM-5 as the catalyst was proposed (Scheme 6), involving the formation of the intermediate peroxide on the Co_3_O_4_ particles encapsulated into the ZSM-5 zeolites, its further decomposition to benzaldehyde and formaldehyde, promoting the continuous conversion of styrene. Benzaldehyde is the major product that then diffuses to the outside of the catalyst. A small part of formaldehyde in solution is oxidized by H_2_O_2_ to form peroxylic acids that react with styrene to generate styrene oxide, which partially hydrolyzes into 1-phenyl-1, 2-ethanediol as the reaction proceeds.

In 2018, metal-loaded ZSM-5 zeolite catalysts were investigated for the selective oxidation of styrene by Hoang et al. [48]. Metal-loaded ZSM-5 zeolites (Cr-ZSM-5 and W-Cr-ZSM-5) were prepared and used for styrene oxidation in the presence of H_2_O_2_. The prepared materials exhibited high catalytic activities for the oxidation of styrene double bonds (Table 6). An excellent styrene conversion (>85% for 6 h reaction at 70 °C) and high selectivity for benzaldehyde (>74%) were observed when using the mesoporous Cr/ZSM-5. Tungsten was loaded on the mesoporous Cr/ZSM-5 particles to enhance the activity of the catalyst. The tungsten-loaded Cr-ZSM-5 showed higher catalytic activity than that of Cr-ZSM-5 for the conversion of fatty acids, although with decreased selectivity to aldehydes.

**Figure 10:**
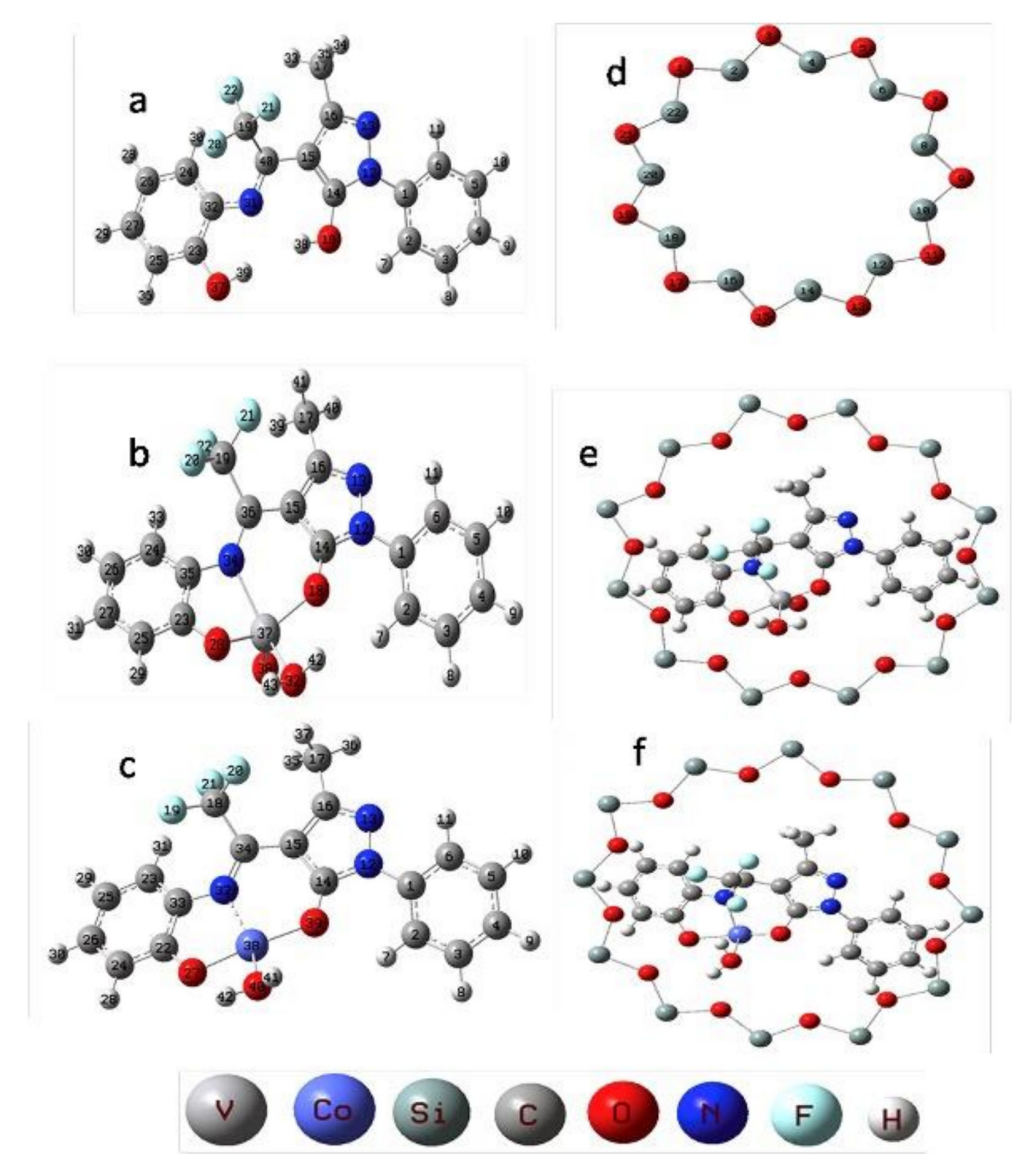
Representative structures for H_2_L ligand (**a**), neat [VO(L)∙H_2_O] complex (**b**), neat [Co(L)∙H_2_O]·H_2_O (**c**), zeolite-Y nanocavity (**d**), [VO(L)∙H_2_O]-Y (**e**) and [Co(L)∙H_2_O]-Y (**f**). Reproduced with permission from ref [49]. Copyright (2017) Elsevier Inc., Amsterdam, The Netherlands.

Modi and coworkers explored the use of zeolite-Y entrapped metallo-pyrazolone complexes as heterogeneous catalysts for the catalytic styrene oxidation reaction [49]. Transition metal [M=VO(IV) and/or Co(II)] complexes with Schiff base ligand, H_2_L, were entrapped in the super cages of zeolite-Y by flexible ligand method (Figure 10). The catalytic performance of these nanohybrid materials was examined in the oxidation of styrene using H_2_O_2_ as an oxidant. The catalytic activity of the entrapped complexes weighed up against the neat complexes (homogeneous system). The effect of experimental variables (such as solvents, the mole ratio of substrate and oxidant, the amount of catalyst and reaction time) was also discussed. Under the optimized reaction conditions, [VO(L)·H_2_O]-Y was found to be the best candidate as a heterogeneous catalyst, achieving 82% conversion of the styrene oxidation reaction.

**Figure 11:**
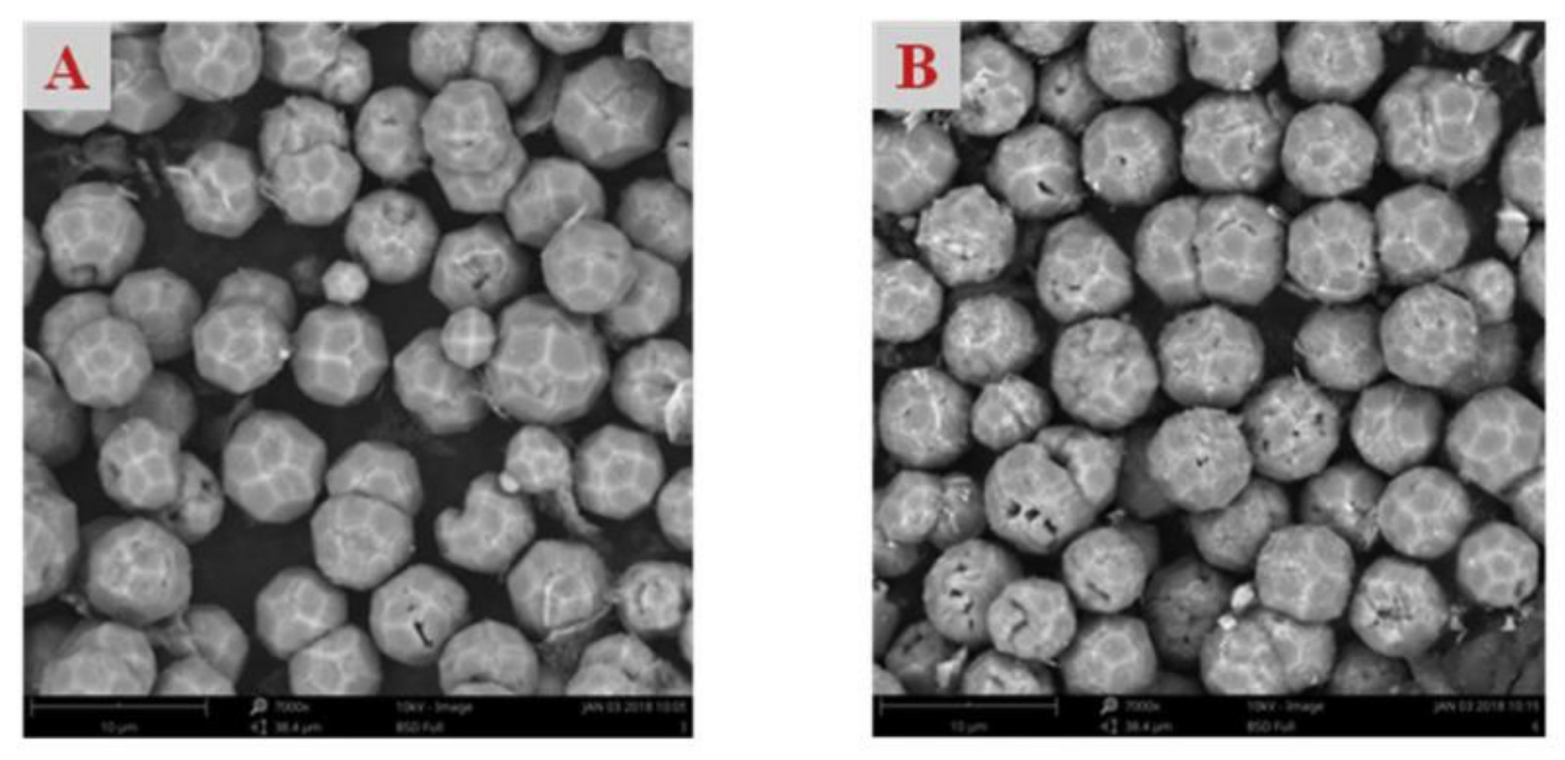
(**A**) SEM image of the analcime zeolite; (**B**) SEM image of Cu/ANA composite material (before reaction). Reproduced with permission from ref [50]. Copyright (2018), Elsevier Inc., Amsterdam, The Netherlands.

In 2019, Gu and coworkers synthesized ANA-zeolite-based Cu nanoparticles (ANA: analcime; NPs: nanoparticles) composite through a hydrothermal method using tetrapropylammonium hydroxide (TPAOH) as a structure-directing agent and explored its catalytic activity [50]. In the Cu@NPs/ANA-zeolite composite catalyst, Cu@NPs were uniformly anchored to the surface of the zeolite carrier (Figure 11).

**Figure 12:**
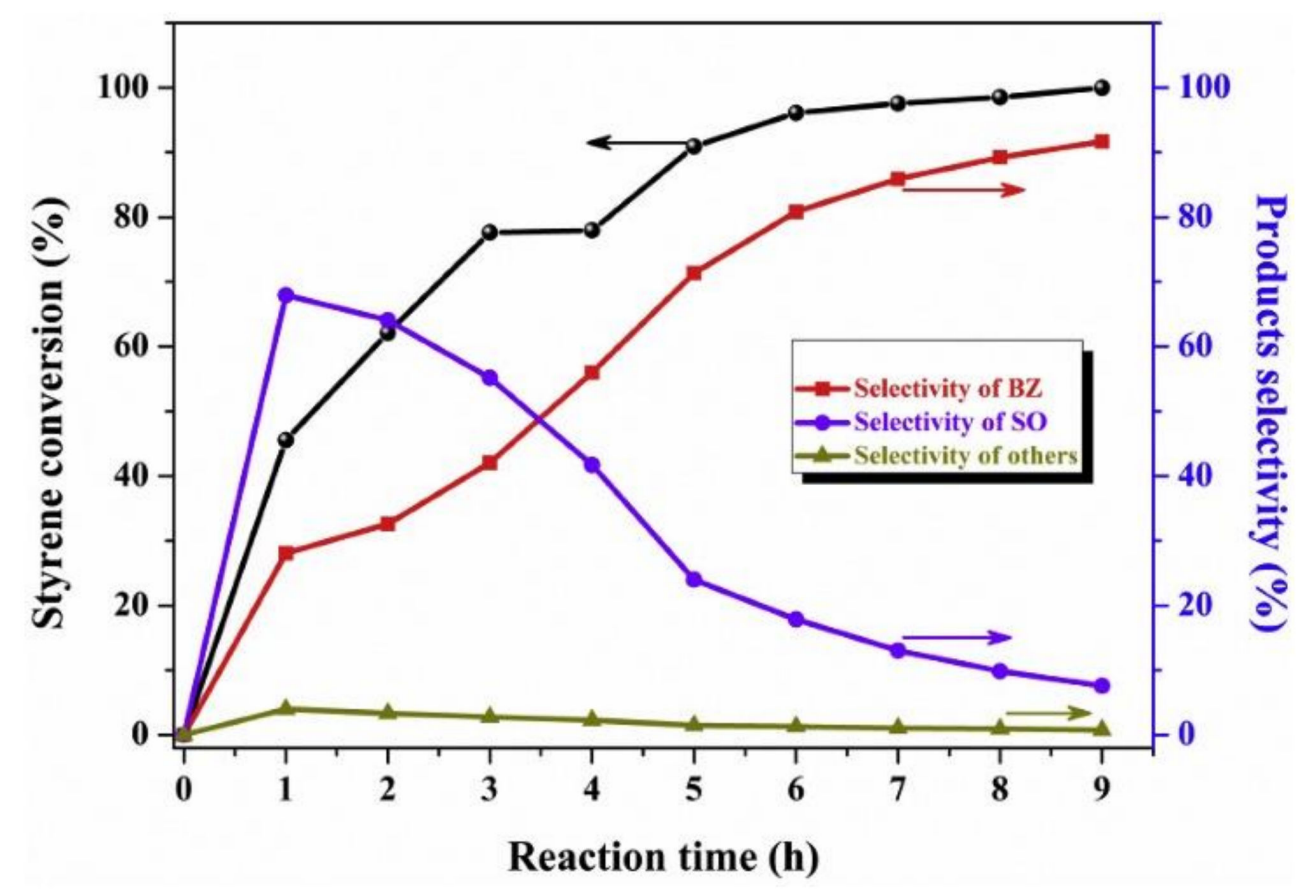
The oxidation of styrene on Cu@nanoparticles (NPs)/analcime (ANA)-zeolite composite catalyst for different times. Reaction conditions: 50 mg catalyst, 1 mL styrene, 5 mL TBHP, 5 mL acetonitrile, stirring speed of 200 r/min, temperature of 80 °C. Reproduced with permission from ref [50]. Copyright (2018), Elsevier Inc., Amsterdam, The Netherlands.

This catalyst exhibited excellent activity and styrene oxidation under optimized reaction conditions (Figure 12). The highest selectivity towards benzaldehyde (90%) was achieved at 80 °C with an almost complete conversion. The solvent played an important role in the oxidation reaction of styrene, and acetonitrile was the more suitable solvent in this case, with 100% styrene conversion and 90% selectivity of benzaldehyde. The catalytic activity of H_2_O_2_ was slightly lower than that of TBHP. Styrene conversion was found to increase with the increase of temperature. In light of the catalytic results, Cu played a significant role in the styrene oxidation reaction. Cu@NPs/ANA composite catalyst was compared with other catalytic systems for styrene oxidation, showing a significantly improved BZ selectivity compared to previous reports in the literature. A more plausible explanation is that the fresh catalyst before reaction contains mainly Cu(0) and Cu(I), and as the recycle reaction runs, Cu(0) and Cu(I) is gradually oxidized by TBHP to Cu(I) and Cu(II). The composite catalyst was recovered and reused in cyclic experiments, with the conversion of styrene for five cycles above 95%.

## 4. Carbon-Based Heterogeneous Catalysts

Carbon materials comprehend a variety of nanomaterials, ranging from activated carbon (AC) to graphite, fullerenes, carbon nanotubes (CNTs), diamond and graphene, etc. Each of these materials presents distinctive features regarding textural parameters and surface chemistry [51,52,53], which makes them remarkably flexible and suitable for several applications, including heterogeneous catalysis. Carbon nanomaterials are attractive and competitive alternatives to be used as catalysts or catalyst supports in several reactions [54,55,56,57,58,59,60,61]. The relevance of its textural parameters as well as its surface chemistry are important factors in achieving good dispersion of the active phase [62,63,64]. The following examples illustrate recent advances in the use of carbon-based materials as heterogeneous catalysts for the selective styrene oxidation to benzaldehyde.

**Figure 13:**
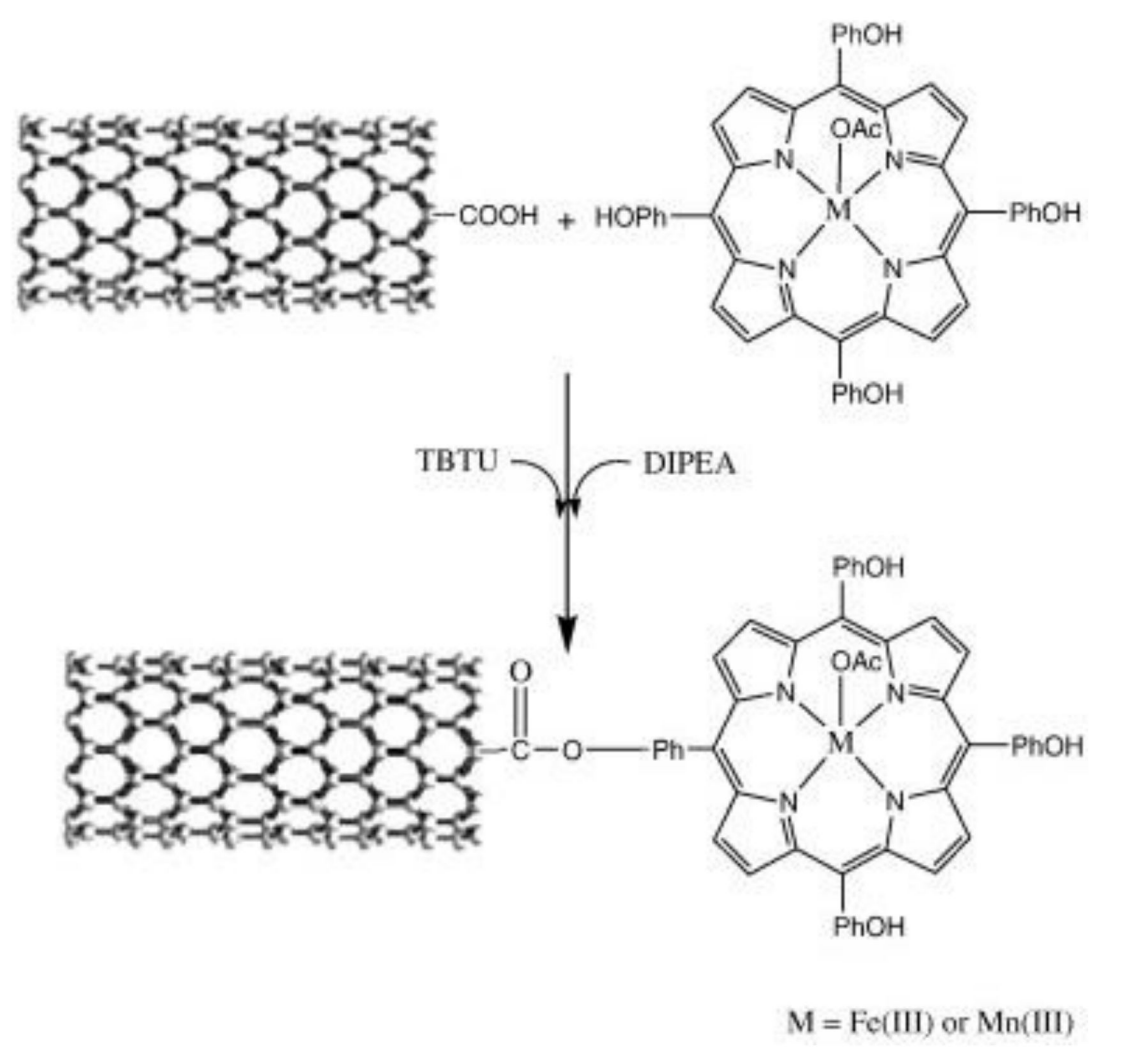
Preparation of multi-wall carbon nanotubes supported metalloporphyrins. Reproduced with permission from ref [65]. Copyright (2012), Elsevier B.V., Amsterdam, The Netherlands.

In 2013, Rayati et al. reported the catalytic activity of CNT@Fe(III) and Mn(III)porphyrins towards styrene oxidation [65]. The preparation of *meso*-tetrakis(4-hydroxyphenyl)porphyrin (H_2_THPP) and Fe(III) porphyrin (FeTHPPCl) and Mn(III) porphyrin (MnTHPPOAc) followed the methods described in the literature [66,67]. The CNT-supported materials were prepared through the covalent bonding between the metalloporphyrins and MWCNTCO_2_H, using MWCNTs with 2 mol% of carboxylic acid groups (Figure 13). Benzaldehyde was observed as the major product in the oxidation of styrene. Higher catalytic activity was observed for the Fe(III) complex (56% conversion with 84% selectivity) comparing to the Mn(III) complex (19% conversion with 79% selectivity). Acetonitrile was the best solvent for the oxidation reaction. The catalytic performance of the manganese porphyrin was more susceptible to the type of solvent. Regarding the reaction mechanism, the formation of allylic products suggests the involvement of a radical pathway. The coordination of TBHP to the metal center of FeTHPPCl-MWCNT or MnTHPP-MWCNT generates a high valent metal–oxo species or an oxo–iron/Mn(IV) porphyrin π-cation radical that performs as the active oxidant species.

The active species transfers an oxygen atom to the double bond, forming styrene oxide via the H abstraction from the allylic positions of the styrene. Unlike FeTHPPCl, which is readily degraded under homogeneous conditions, the CNT-supported material was reused four times without losing its catalytic activity.

The presence of AC, porous carbon materials, in the selective oxidation of styrene to benzaldehyde catalyzed by *p*-toluenesulfonic acid (*p*-TsOH), was investigated by Li et al. [68]. The authors investigated the influence of several reaction parameters, as reaction time, temperature, catalyst mass, and styrene/H_2_O_2_ molar ratios, in the catalytic activity. A strong promoting effect of AC on the *p*-TsOH/H_2_O_2_ system was found as well as the influence of AC and *p*-TsOH ratios in the catalytic results.

**Scheme 7:**
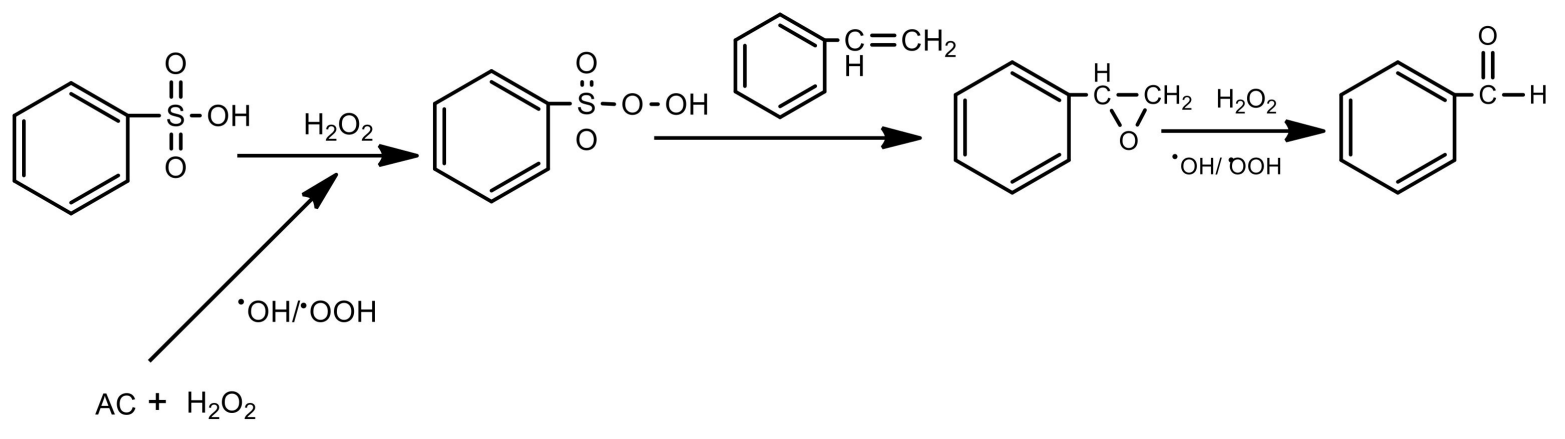
Oxidation of styrene in the *p*-TsOH/HAC/H_2_O_2_ system [68].

The reduction of oxygen-containing groups (–OH,–CO_2_H) on AC by a high-temperature treatment or introduction of–SO_3_H groups onto AC, generating HAC, also affected the styrene conversion, with the latter being more effective in promoting styrene oxidation. Both *p*-TsOH and HAC presented very low activity for styrene oxidation, but when *p*-TsOH and HAC was used simultaneously, the styrene conversion was eight times higher than that using either *p*-TsOH or HAC, while the selectivity for benzaldehyde only showed only a moderate decrease. The promotion mechanism of HAC in this system was ascribed to the interaction of the radical reaction (HAC/H_2_O_2_) and non-radical process (*p*-TsOH/H_2_O_2_) (Scheme 7).

**Figure 14:**
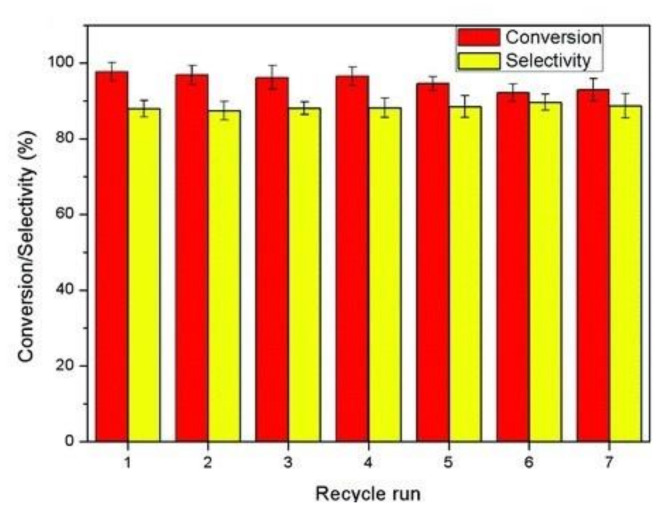
Recycling of the catalyst VC-3. Reproduced with permission from ref [69]. Copyright (2018), The Royal Society of Chemistry, Amsterdam, The Netherlands.

In 2018, Zou et al. explored the catalytic performance of vanadium pentoxide-modified graphitic carbon nitride (V_2_O_5_/*g*-C_3_N_4_) composites in the oxidation of styrene [69]. The excellent performance of *g*-C_3_N_4_ materials as catalysts is anticipated given their resemblance to graphene, which confers their high thermal and chemical stability, with a simply tailorable structure [70]. The V_2_O_5_/*g*-C_3_N_4_ composites were prepared with different weight ratios (1–5%) of V_2_O_5_ loads, obtained through a wet impregnation method and calcination and denoted as VC-1 to VC-5 according to the V_2_O_5_ load. These materials were investigated as catalysts for the oxidation of styrene, as well as bare V_2_O_5_ and *g*-C_3_N_4_, studied for comparison. The VC-3 composite showed the best catalytic performance for the styrene oxidation reaction, obtaining 88% selectivity to benzaldehyde at 99% of styrene conversion under visible light irradiation. Other styrene substrates were also selectively converted to the corresponding aldehydes, achieving high yields (up to 92%) using this catalytic system. The recyclability of the catalyst was assessed through a series of seven reuse cycles using VC-3 as a catalyst under optimal conditions (Figure 14), without an obvious decrease of activity. A mechanism was postulated for the oxidation reaction with H_2_O_2_, following a •OH radical pathway.

**Scheme 8:**
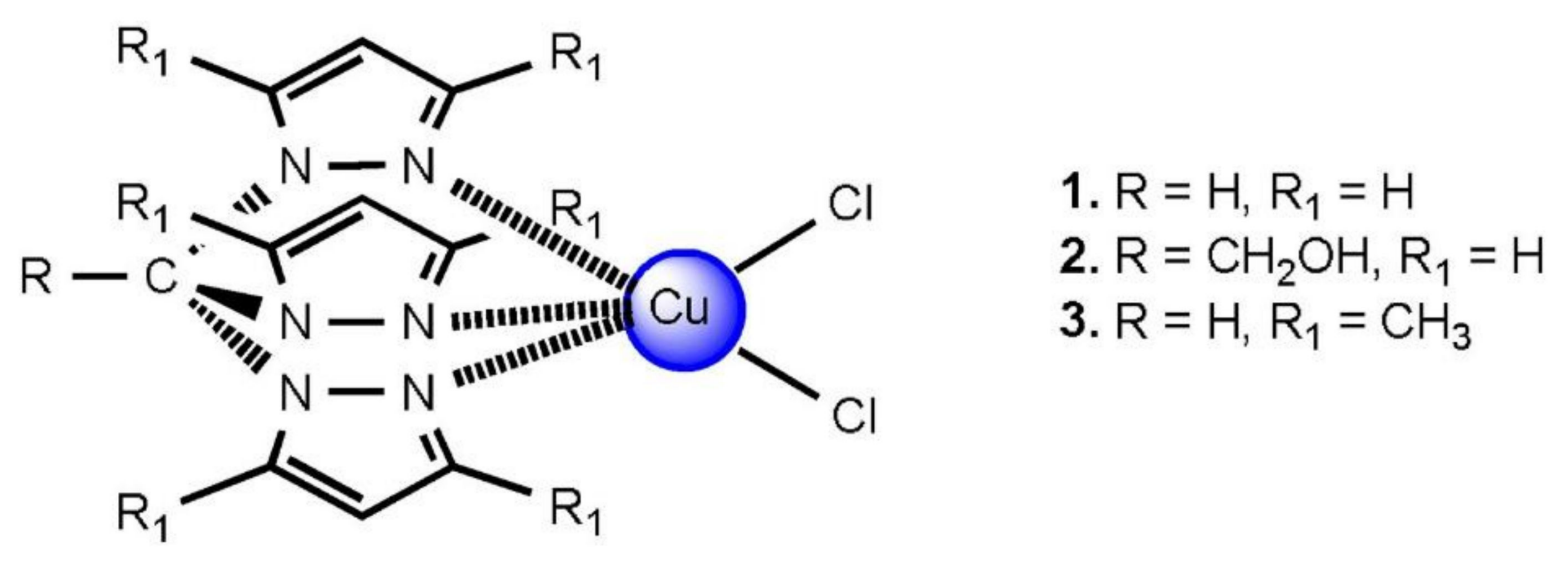
Mononuclear half-sandwich C-homoscorpionate copper(II) complexes catalysts for styrene oxidation:[CuCl_2_{κ^3^-HC(pz)_3_}] (1), [CuCl_2_{κ^3^-HOCH_2_C(pz)_3_}] (2) and the new [CuCl_2_{κ^3^-HC(3,5-Me_2_pz)_3_}] (3) [71].

In 2020, Duarte et al. reported the catalytic activity of mononuclear C-scorpionate Cu(II) complexes [CuCl_2_{κ^3^-RC(R_1_-pz)_3_}[pz = pyrazol-1-yl; R = R_1_ = H, R = CH_2_OH, R_1_ = H and R = H, R_1_ = 3,5-Me_2_] using sucrose-derived hydrochars as supports (Scheme 8) [71]. The coordinative versatility displayed by C-homoscorpionate ligands has led in recent years to the formulation of active and selective catalysts for several oxidation reactions, in particular of alkanes [72,73], alcohols [74] and ketones [75], and, very recently, for the homogeneous oxidation of styrene under mild conditions [76].

In contrast to other widely used carbon materials, hydrochars, being synthesized by hydrothermal carbonization of several types of biomass, were not yet quite explored as catalytic supports. While in homogeneous conditions, a benzaldehyde yield above 60% and 100% selectivity, with a TOF of 1.3 × 10^4^ h^−1^ were observed, the use of hydrochars as support enabled to obtain solvent-free catalytic systems for styrene oxidation. Although the heterogeneous catalytic system has provided slightly lower benzaldehyde yields, up to 52%, these were obtained after only 5 min of reaction (Table 7). The immobilization of the mononuclear C-scorpionate Cu(II) complexes at functionalized also required considerably lower amounts of oxidant than in the homogeneous system. The influence of the properties of the scorpionate as well as of other operational parameters on the catalytic performance of the above systems were addressed.

**Figure 15:**
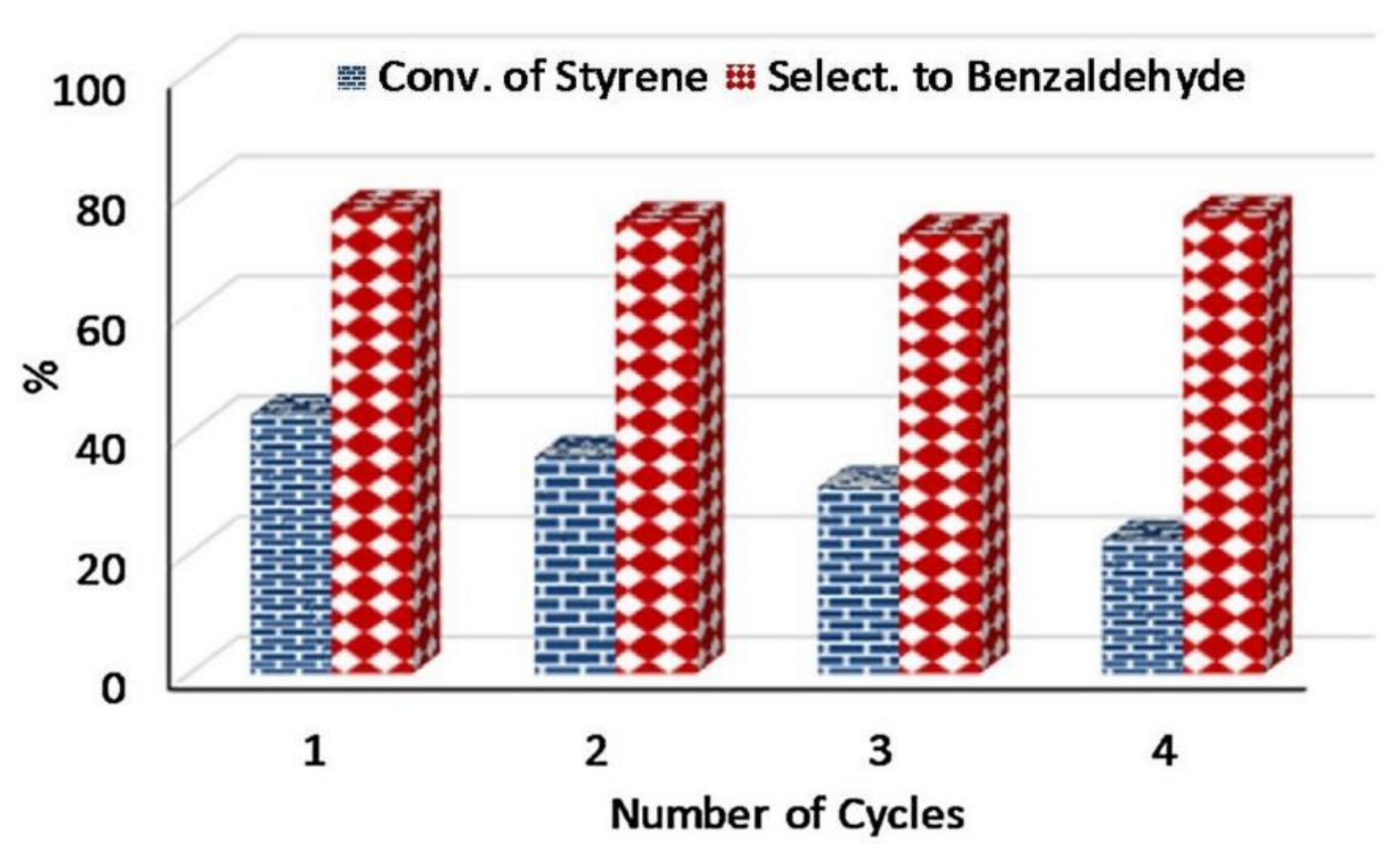
Recycling experiments for catalyst 1@S0_Na. Reproduced with permission from ref [71]. Copyright (2019), Elsevier B.V., Amsterdam, The Netherlands.

Upon optimization of the reaction parameters, the best results were obtained for solvent-free and low amounts of oxidant in homogeneous conditions. The conversion of styrene increased with the amount of oxidant under heterogeneous conditions, with a selectivity decrease for benzaldehyde, due to an increase of overoxidation products. These materials were the first recyclable tris(pyrazol-1-yl)methane catalysts reported to date for the selective oxidation of styrene to benzaldehyde. Sample 1@S0_Na was chosen to perform the recycling tests (Figure 15), and selectivity toward benzaldehyde was maintained after four consecutive cycles.

**Figure 16:**
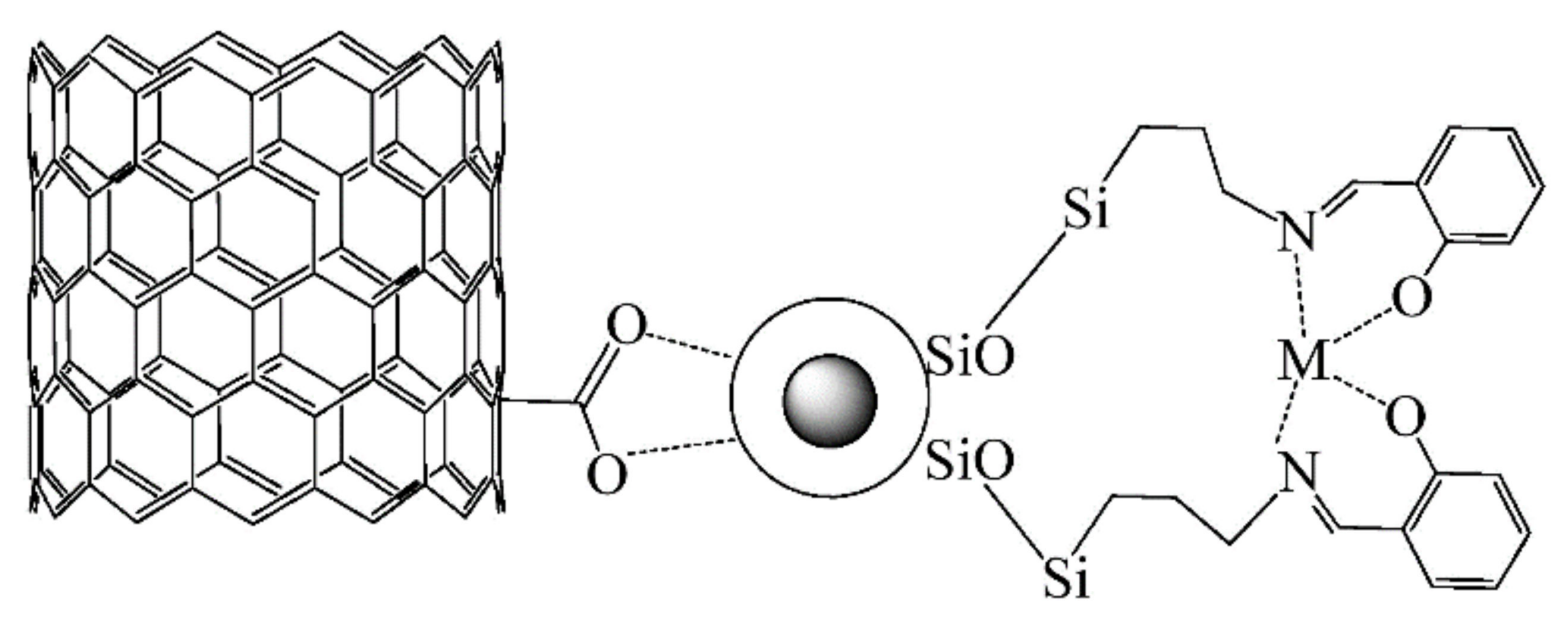
Preparation of M(II)/Schiff base@MWCNT-Fe_3_O_4_/SiO_2_ (M=Co, Cu). Reproduced with permission from ref [77]. Copyright (2019), Elsevier Masson SAS, Amsterdam, The Netherlands.

Lashanizadegan and coworkers performed the immobilization of Cu(II) and Co(II) Schiff base complexes on functionalized MWCNTs and investigated their catalytic activity towards the selective oxidation of styrene [77]. Cu(II) and Co(II) Schiff-base complexes were anchored on magnetized MWCNTs (Cu(II)/Schiff base@MWCNT-Fe_3_O_4_/SiO_2_ and Co(II)/Schiff base@MWCNT-Fe_3_O_4_/SiO_2_) (Figure 16). The synthesized nanostructures were applied as catalysts in the oxidation of styrene, among other substrates. The catalytic results revealed that even though Cu(II)/Schiff base@MWCNT-Fe_3_O_4_/SiO_2_-catalyzed styrene oxidation reaction in the presence of TBHP, yielding styrene oxide (73%) with 94% conversion when Co(II)/Schiff base@MWCNT-Fe_3_O_4_/SiO_2_ was used in the presence of H_2_O_2_ and imidazole, 67% selectivity to benzaldehyde was achieved with 100% conversion. The catalyst was easily recovered utilizing an internal magnet and reused in several cycles without significant activity loss.

**Scheme 9:**
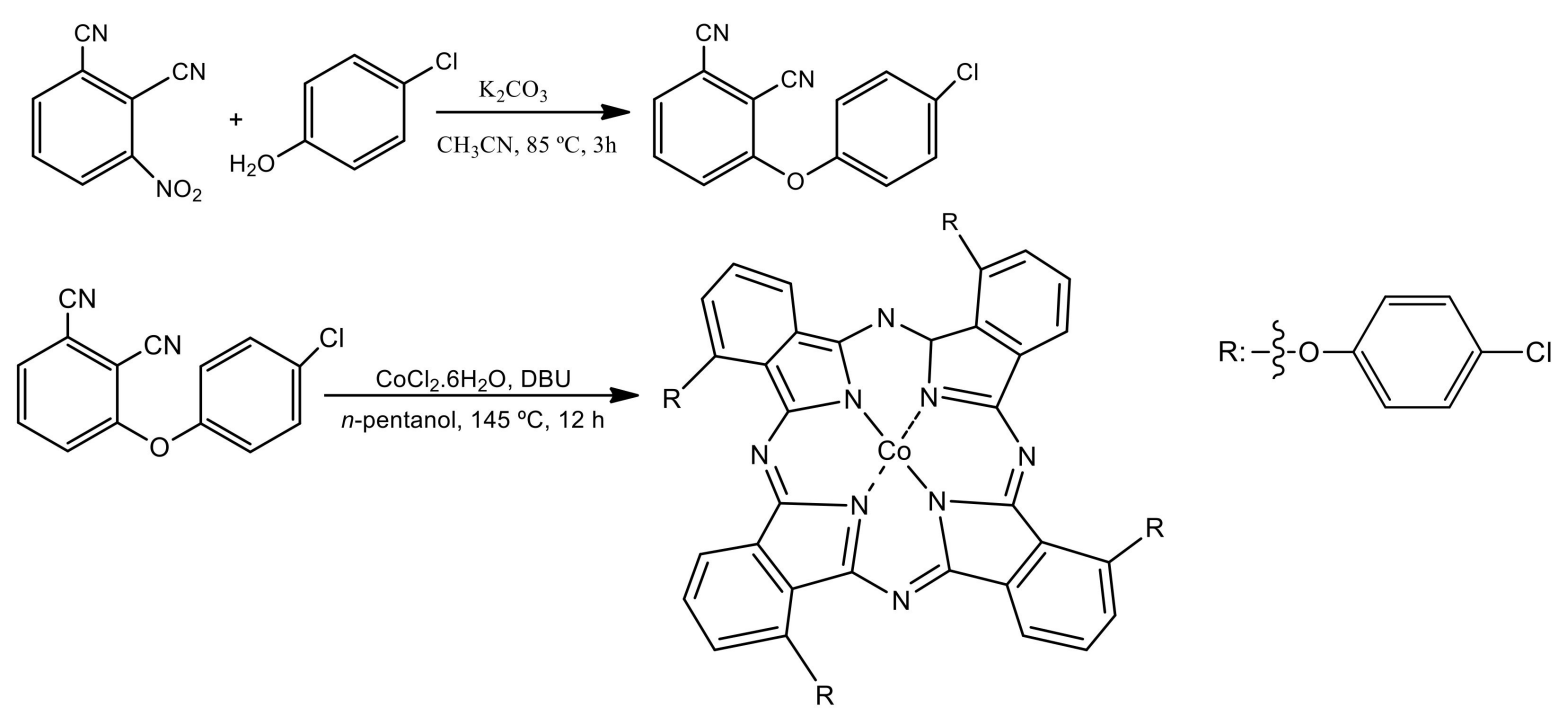
Synthetic route for the preparation of 4a(OPh-*p*-Cl)CoPc [78].

MWCNTs were also used as support for cobalt Pc- (Pc: phthalocyanine) composites on the oxidation of styrene to benzaldehyde by Wang et al. [78]. The authors prepared novel tetrasubstituted cobalt Pc-MWCNT composites ([4a(OPh-*p*-Cl)CoPc]-MWCNTs), used as heterogeneous catalysts for the catalytic oxidation of styrene to benzaldehyde (Scheme 9).

**Figure 17:**
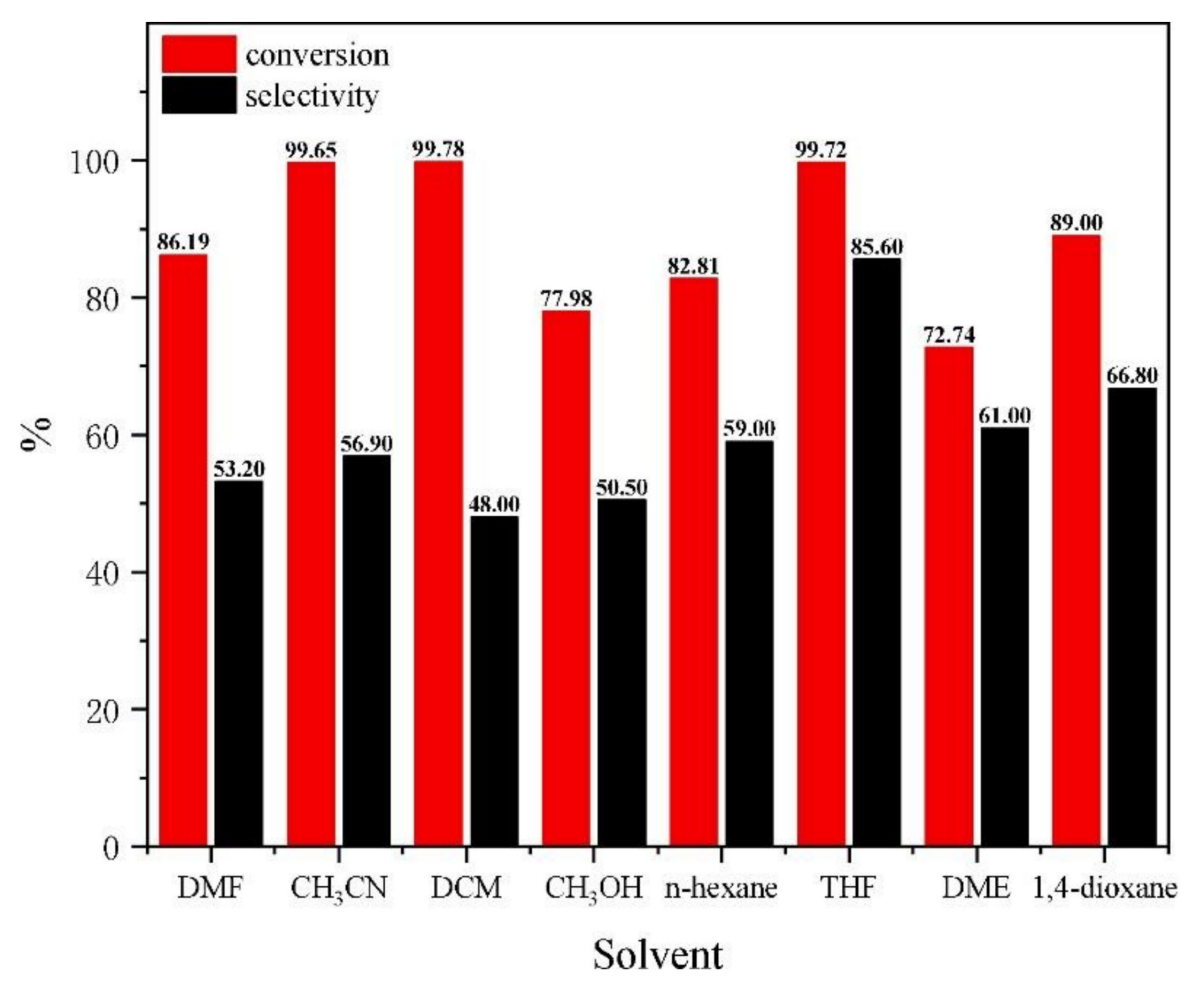
Effect of different solvents on catalytic conversion and selectivity. Reproduced with permission from ref [78]. Copyright (2020), Elsevier Masson SAS, Amsterdam, The Netherlands.

The authors studied the effect of solvent, oxidant, catalyst amount, temperature and reaction time on the catalytic activity. The [4a(OPh-*p*-Cl)CoPc]-MWCNTs composites displayed a very high catalytic performance, achieving the highest styrene conversion of 99% and benzaldehyde selectivity of 89% under optimized reaction conditions (Figure 17). Moreover, the authors also postulated the plausible reaction mechanism for the selective styrene oxidation by [4a(OPh-*p*-Cl)CoPc]-MWCNTs composites to benzaldehyde. The intrinsic π-π conjugated structure of Pcs leads to the strong agglomeration effect between molecules, seriously affecting the catalytic activity of Pcs. The features of MWCNTs allowed a uniform load of Pcs by an ultrasonic method, significantly reducing the agglomeration effect of Pcs through an increase of the area of the catalytic surface.

In 2020, Saini and colleagues used waste printer ink to produce magnetically separable iron oxide-doped nanocarbons and applied it to styrene oxidation [79]. This simple, cost-effective method for the isolation of magnetically separable self-doped iron oxide nanocarbons’ (FeO-NC) from a carbon source material involved the carbonization of waste printers. The as-prepared FeO-NC was investigated as a heterogeneous catalyst for the oxidation of styrene to benzaldehyde, obtaining 70% yield. Although the reaction was performed under solvent-free conditions, the authors also explored various polar/nonpolar solvents, achieving good results with nonpolar solvents, achieving 68% for toluene, as well as in polar aprotic solvents, i.e., 1,4-dioxane and N-methyl-2-pyrrolidone, obtaining benzaldehyde yields of 70 and 72%, respectively. Importantly, the reaction could also be performed in water, producing a considerable yield of 65% of the desired product. Significantly, FeO-NC were magnetically separable, presenting good recyclability, endorsing the sustainability of the process.

## 5. Silica-Based Heterogeneous Catalysts

The potentialities of silicas as supports for metal catalysts in numerous reactions as oxidation, alkylation and hydrogenation have been widely recognized [80]. These hybrid mesoporous materials present as major advantages extremely high surface areas and free accessibility of their porous networks [81,82]. Their well-ordered structure with tunable porosity as well as the abundance of surface silanol groups enables its covalently mediated functionalization [83]. Other research topics rely on the role of the silica structure as support, mesopore size enlargement to optimize the catalytic performance. The following examples account for recent contributions on this field, exploring the use of silica-based materials, such as SBA-15, MCM-41, MCM-48 or mesoporous SiO_2_.

**Scheme 10:**
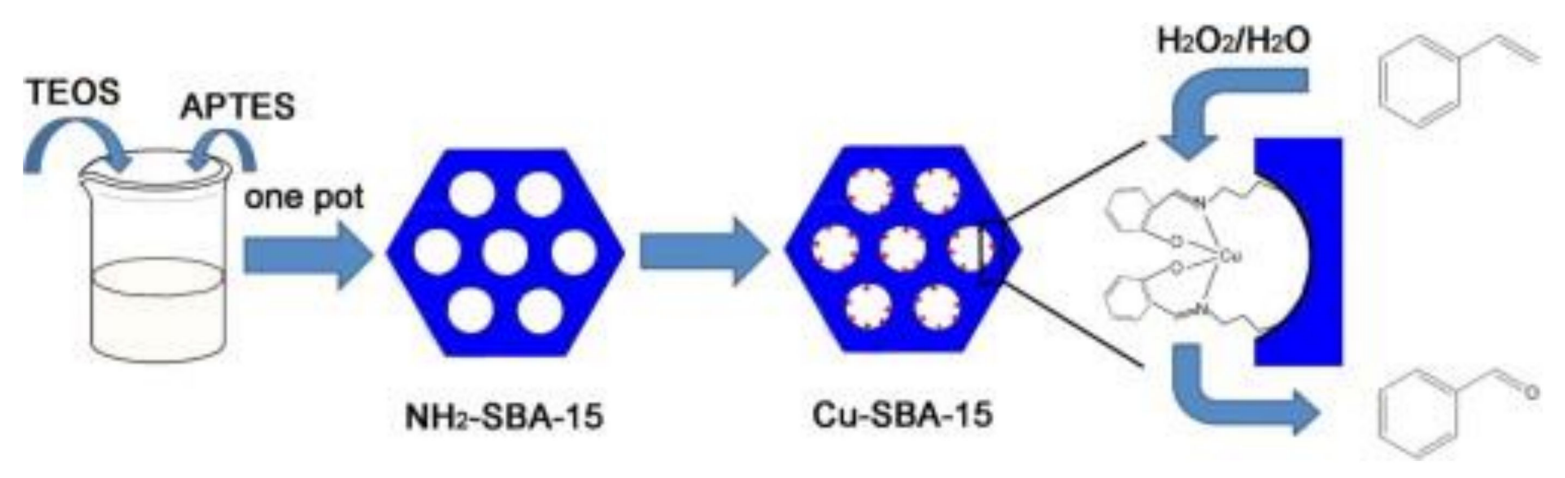
Catalytic oxidation of styrene to benzaldehyde over a copper Schiff-base/SBA-15 catalyst. Reproduced with permission from ref [84]. Copyright (2014), Elsevier B.V., Amsterdam, The Netherlands.

Zhu et al. explored the catalytic oxidation of styrene to benzaldehyde over a copper Schiff-base/SBA-15 catalyst (Scheme 10) [84]. The authors aimed to overcome the difficult separation of the high catalytic performing metal Schiff-base complexes. The amino-modified SBA-15 (NH_2_-SBA-15) mesoporous material was obtained through co-condensation of tetraethyl-orthosilicate with 3-aminopropyltriethoxysilane in the presence of a pore-directing agent under acidic conditions. The SBA-15-supported Cu Schiff-base complex (Cu-SBA-15) was synthesized by condensation of salicylaldehyde with NH_2_-SBA-15, followed by the addition of a solution of Cu(NO_3_)_2_. The prepared complex was thoroughly characterized and used for the selective oxidation of styrene to benzaldehyde.

A maximum styrene conversion of 84% was achieved for styrene with 84% selectivity for benzaldehyde when the reaction was performed with a 2:1 molar ratio of oxidant: a substrate with a 3.8 wt % catalyst load at 100 °C for 8 h. The catalyst was reused three times without a considerable loss of activity. The authors pointed out the contribution of the high specific surface area, uniformly sized pore channels, and well-distributed active centers of the catalyst for the enhanced catalytic activity observed. The main product was benzaldehyde, and no styrene oxide was detected.

Recycling studies were conducted for Cu-SBA-15 (Table 8), and an insignificant decrease in selectivity for the recovered catalyst was observed, most likely attributed to the presence of residual organics on the surface of the catalyst hindering the desorption of benzaldehyde from the catalytic centers; therefore, leading to over-oxidation to benzoic acid.

**Scheme 11:**
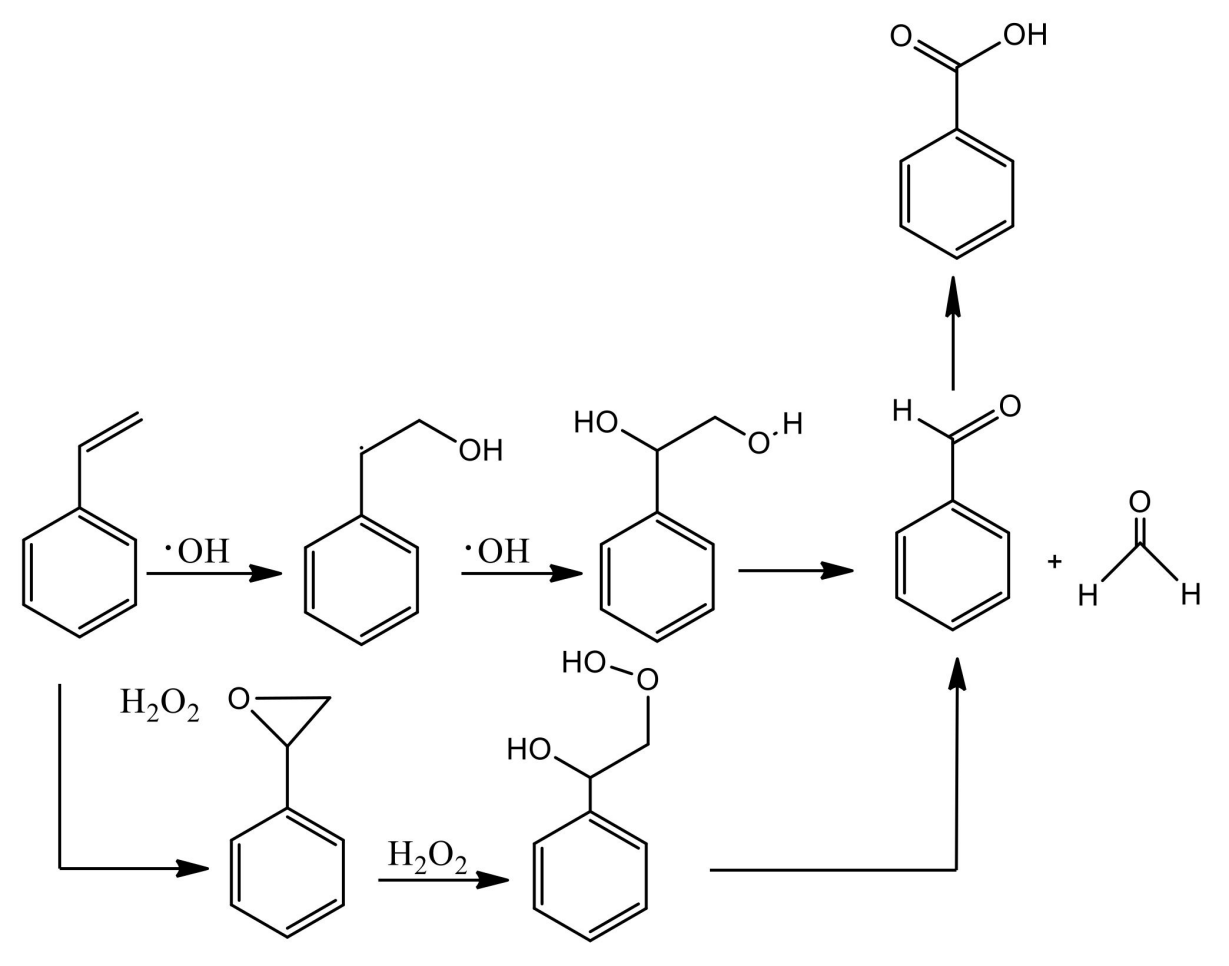
Proposed reaction mechanism of styrene oxidation [84].

The authors concluded that styrene oxidation to benzaldehyde over the prepared copper(II) Schiff-base complexes follows a pathway involving a radical mechanism since styrene oxide was not detected during the course of the reaction [85]. In addition, 2 equiv. of H_2_O_2_ were required for styrene oxidation to benzaldehyde, in good agreement with the experimental results, showing low conversion when less than 2 equiv. of H_2_O_2_ was used (Scheme 11).

**Scheme 12:**
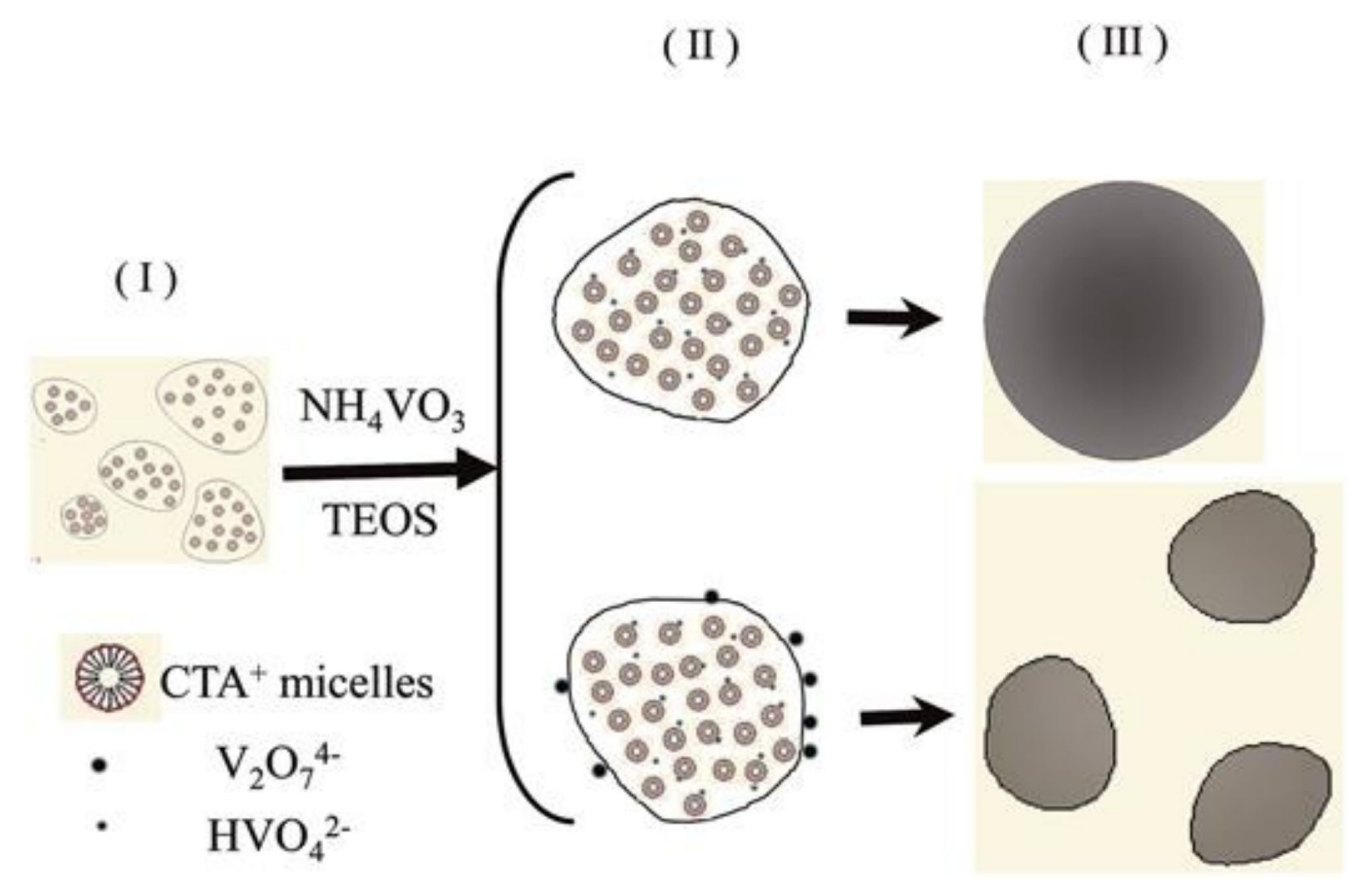
The proposed formation mechanism of the spherical VMCM-48 based on the Stöber process. (I) The micelles are produced from the template solution (step I); (II) both the TEOS and vanadium sources hydrolyze to form disordered clusters; (III) cluster growth into large and packed particles. Reproduced with permission from ref [86]. Copyright (2014), The Royal Society of Chemistry, Amsterdam, The Netherlands.

A series of spherical, mesoporous V-MCM-48 catalysts presenting different V/Si atomic ratios were prepared by Wang et al. via a direct hydrothermal method [86]. The V-MCM-48 samples showed a homogeneous dispersion and highly ordered cubic mesostructure. The authors proposed the possible formation mechanism of the spherical V-MCM-48 based on the Stöber process (Scheme 12).

Characterization results demonstrated that most of the vanadium is present as tetracoordinate V^4+^ species in a silicate framework, with only a small amount existing as isolated V^5+^ species on the surface. The catalytic activity of the prepared materials was evaluated in the oxidation of styrene to benzaldehyde using H_2_O_2_ as oxidant, exhibiting high catalytic activity (Table 9). The spherical morphology significantly contributed to the enhanced catalytic performance observed for these materials. V^5+^ species on the surface and/or originated from the oxidation of V^4+^ in the framework act as active sites. Even though the catalytic performance of materials was correlated to the number of active vanadium species, the spherical morphology also contributed to the accelerated mass transport. The conversion of styrene and the selectivity to benzaldehyde were 83 and 91%, respectively, under optimized reaction conditions.

In 2015, Li and coworkers selected SBA-15 and MCM-41 for the preparation of Co-SBA-15 and Co-MCM-41 mesoporous catalysts using an ultrasonic-assisted “pH-adjusting” technique [87], a fast and cost-effective method for the preparation of mesoporous silica materials.

**Figure 18:**
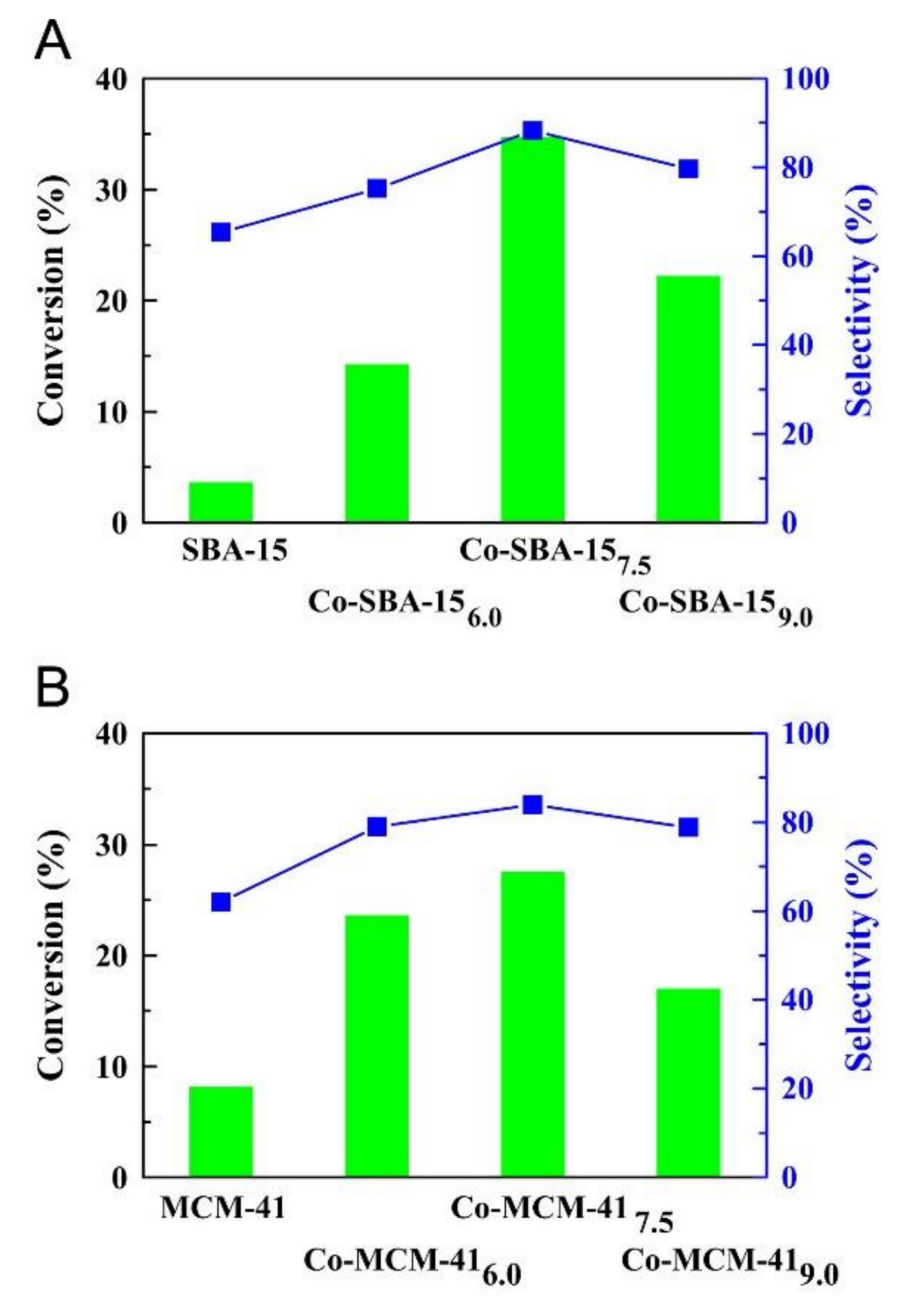
Effect of reaction time on oxidation of styrene with H_2_O_2_ over Co-SBA-15 7.5 (**A**) and Co-MCM-41 7.5 (**B**) catalysts. (bar) Styrene conversion, (□) benzaldehyde selectivity. Reaction condition: styrene, 10 mmol; H_2_O_2_, 10 mmol; acetonitrile, 10 mL; catalyst,0.05 g; temperature 70 °C. Reproduced with permission from ref [86]. Copyright (2015), The Royal Society of Chemistry, Amsterdam, The Netherlands.

The pH values used considerably affected the Co content, as well as the textural parameters of the final catalyst. The physicochemical structures of the materials were thoroughly characterized, and a correlation was established with their catalytic activity towards styrene oxidation. The catalytic performance of various Co-based catalysts showed that for pure SBA-15, only 4% of styrene conversion was achieved. The nature of cobalt species depended on the type of mesoporous silica as well as pH values. The highest conversion of styrene of 35% with 88% selectivity to benzaldehyde was obtained for Co-SBA-15 catalyst at pH = 7.5, at the mole ratio of 1:1 (styrene to H_2_O_2_) at 70 °C (Figure 18).

The difference in the catalytic performance of Co-SBA-15 and Co-MCM-41 catalysts is most likely due to the different cobalt species homogeneously incorporated into the framework of the materials. For Co-SBA-15 in single-site Co(II) state was observed, while Co_3_O_4_ particles were loaded on Co-MCM-41 catalysts. The catalytic tests showed that the single-site Co(II) state incorporated into the framework of SBA-15 was more advantageous for the catalytic oxidation of styrene.

**Scheme 13:**
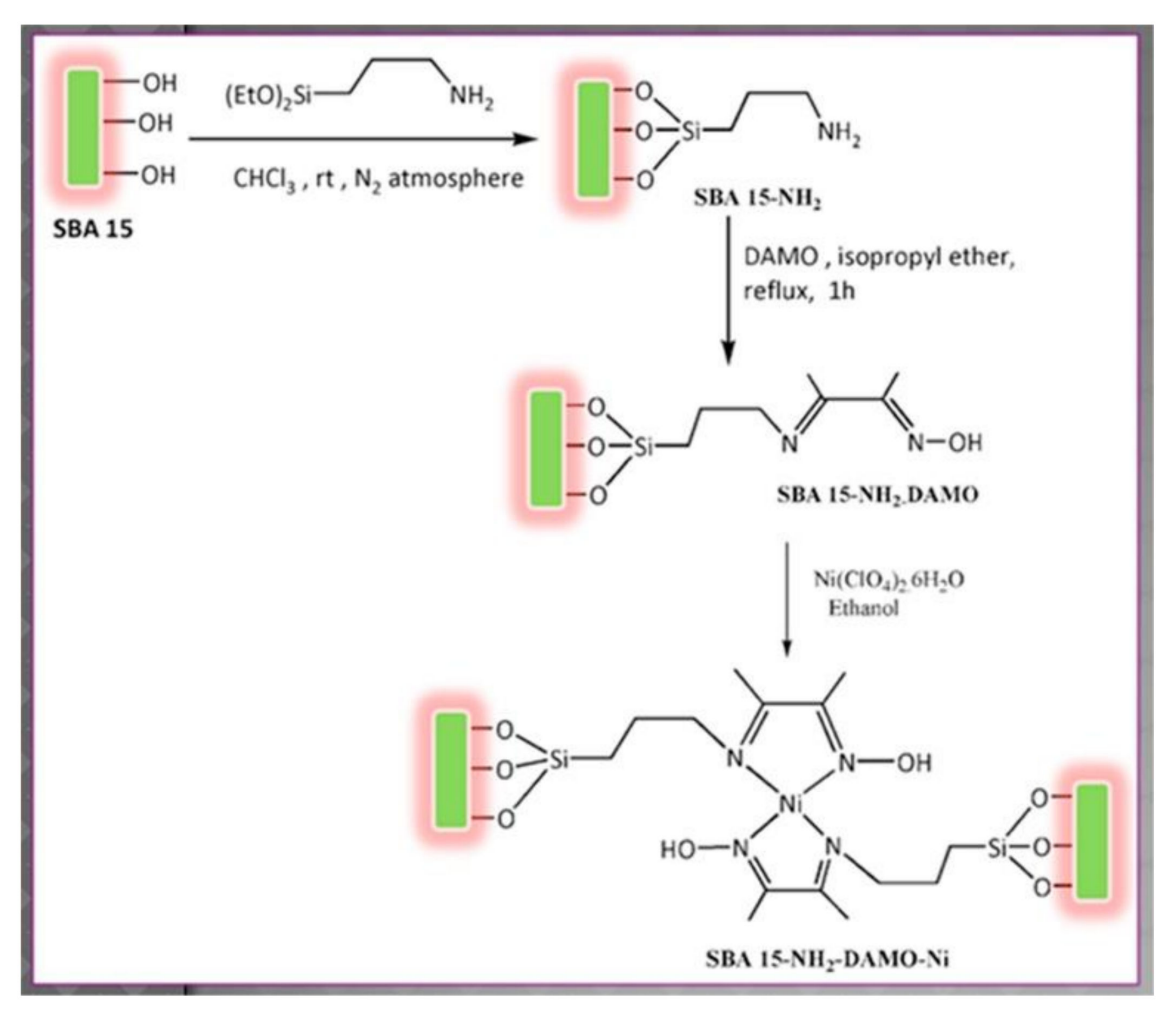
Route to synthesize functionalized SBA-15 supported nickel(II)-oxime–imine catalysts. Reproduced with permission from ref [88]. Copyright (2016), Elsevier Inc., Amsterdam, The Netherlands.

In 2016, Paul and coworkers reported the catalytic styrene liquid phase oxidation over functionalized SBA-15 supported nickel (II)–oxime–imine under solvent-free conditions [88]. This novel oxime–imine highly ordered mesoporous SBA-15 (SBA-15-NH_2_-DAMO) was synthesized via a postsynthesis functionalization with 3-aminopropyl-triethoxysilane (APTES) followed by the Schiff base condensation with diacetyl mono-oxime, which was further reacted with Ni(ClO_4_)_2_ yielding the catalyst SBA-15-NH_2_-DAMO-Ni (Scheme 13).

The heterogeneous styrene oxidation to benzaldehyde was carried out over SBA-15-NH_2_-DAMO-Ni using TBHP, achieved 85% conversion, with 89% selectivity to benzaldehyde. Radical trap experiments revealed that the catalytic oxidation of styrene proceeds through a concerted mechanism. Under homogeneous conditions with a Schiff base ligand-derived complex, a total conversion of 35% was observed. The catalytic oxidation of styrene using H_2_O_2_ showed very poor conversion (4%), most likely due to the decomposition of the Schiff base part of the catalyst, as H_2_O_2_ is a stronger oxidant than TBHP. The catalyst was reused for 5 consecutive runs without significant degradation.

The role of silica as support for bi- and trimetallic catalysts on the heterogeneous catalytic oxidation of styrene was explored by Parehk et al. [89]. Silica-supported bimetallic and trimetallic transition metal catalysts presenting different metal loads (5 to 30 wt %) of Cu, Ni and Co were prepared via an ultrasonic cavitation-impregnation method [90]. Important changes in textural parameters were found for the bimetallic and trimetallic catalyst samples. As the metal loading increases from 10 to 30 wt %, an increase in the size of the catalyst particles was observed due to the agglomeration of the particles. The better metal dispersion was obtained for the catalyst with the lowest metal loading, 10CuNiCoSiO, showing little agglomeration.

The catalytic activity of the prepared materials, silica-supported CuNiSiO, NiCoSiO and CuCoSiO, were investigated in the liquid phase oxidation of styrene, using TBHP, under mild conditions. The tested catalysts achieved 62, 51 and 64% styrene conversion, respectively, with selectivities of benzaldehyde in the range of 56–66%. The trimetallic catalytic systems showed higher activity as compared to the bimetallic systems, even the 5CuNiCoSiO trimetallic catalyst with a total metal loading lower than in the bimetallic catalysts. The good catalytic activity and selectivity are most likely due to a good metal dispersion and a cooperative effect produced by the mixed metals on the mesoporous support with the high surface area. The metal-support interaction improves the oxygen transfer capacity and availability, enhancing the rate of the oxidation reaction. For the best performing trimetallic catalyst, 30CuNiCoSiO, 95% of styrene conversion with 74% benzaldehyde selectivity under optimum conditions, was obtained, comparing favorably to all the metals and metal oxides supported on different supports reported in the literature. The 10CuNiSiO catalyst was successfully reused three times without loss of their catalytic activity and no signs of metal leaching (Table 10).

THBP was a better oxidant compared to H_2_O_2_ for this system, and the best catalytic performance was obtained in acetonitrile, possibly due to a higher solubility of all reagents. Synergistic effects were observed experienced among all the metals being responsible for the enhancement of the catalytic activity in the case of the trimetallic catalysts. Furthermore, these catalysts showed high yields towards benzaldehyde when compared to other catalysts with precious metal catalysts (Au and Ag).

In 2016, Sun and Hu prepared mesoporous catalysts based on SBA-15-functionalized materials with controllable H_3_PW_12_O_40_ (PW_12_) loaded using a one-step co-condensation method and investigated their catalytic performance towards the oxidation of styrene to benzaldehyde in the presence of H_2_O_2_ [91]. POMs are catalysts for oxidation reactions to environmentally friendly oxidants, although the bulk form of these materials lacks efficiency given their low surface area and a limited number of acid sites. POMs are also readily soluble in many solvents, disabling their complete recovery and recycling. The authors observed an increase in the specific surface area of the active components upon the incorporation of PW_12_ into the mesoporous network of SBA-15. A homogeneous dispersion of the Keggin units with lower loadings in the composites was also observed. PW_12_/SBA-15 materials displayed catalyst particle sizes in the nanometers range, and an ordered mesostructure of PW_12_/SBA-15 was further confirmed by TEM. The key reaction parameters were studied, including PW_12_ loading, reaction temperature, solvent, oxidant amount, and reaction time on the catalytic performance of the materials applied to styrene oxidation. The catalytic activity of PW_12_/SBA-15-x (x = 8.9, 14.5, and 15.7) catalysts, SBA-15, and PW_12_ were compared (Table 11). The SBA-15 support was inactive for styrene oxidation, and for neat PW_12,_ a benzaldehyde yield of only 7.1% was achieved, in spite of its highest density of Brønsted acid sites. Regarding the PW_12_/SBA-15-x catalysts, an increase in styrene conversion was observed with the PW_12_ content. A large number of PW_12_ active sites increased the decomposition rate of H_2_O_2_. However, PW_12_/SBA-15–15.7 demonstrated a small change in conversion of styrene compared with PW_12_/SBA-15–14.5, suggesting that at higher PW_12_ loading, a mesopore blockage can occur, causing oxidative sites to be inaccessible during the reaction. The heterogeneously catalyzed styrene oxidation showed that benzaldehyde was the only product.

**Figure 19:**
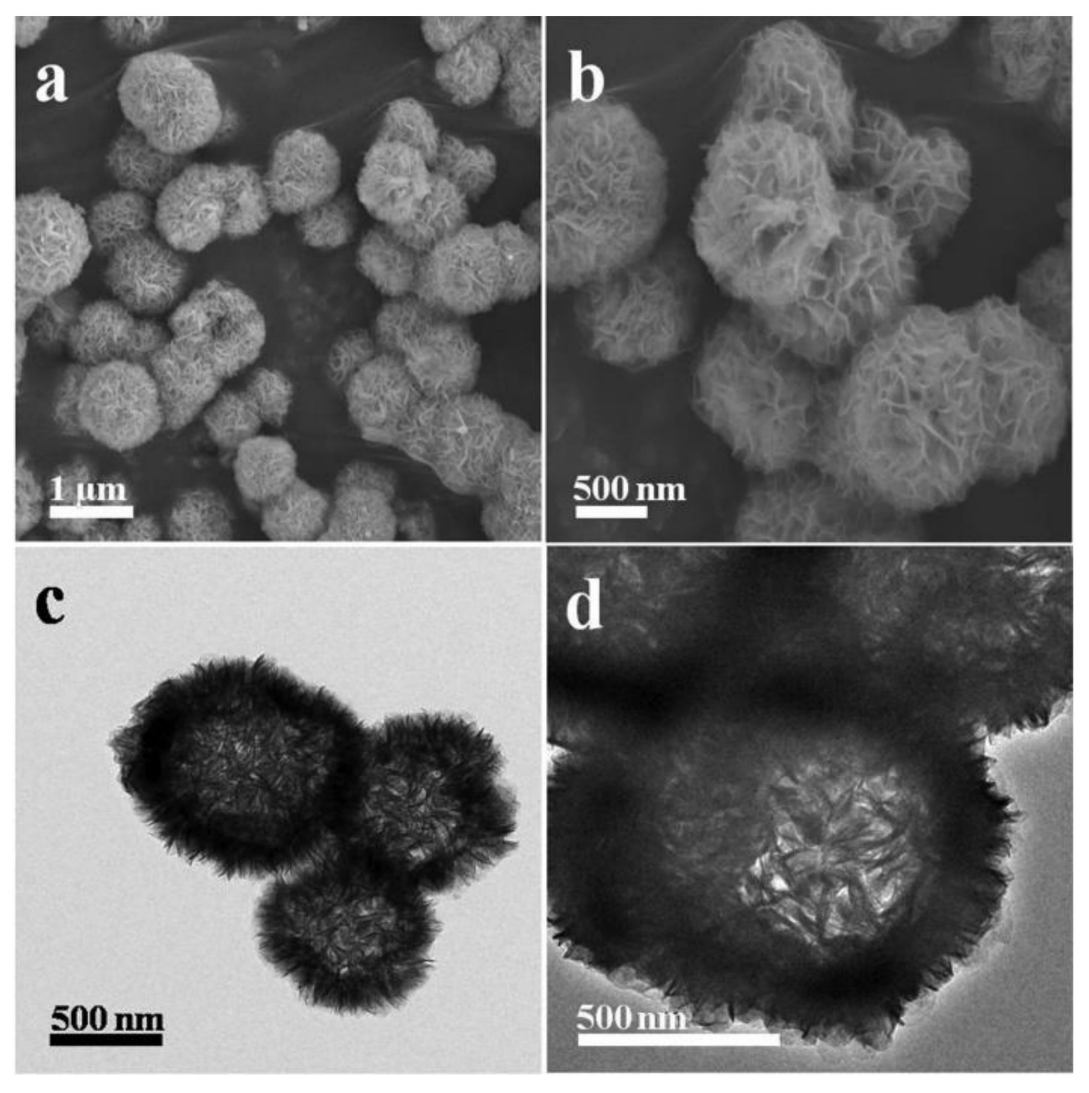
Representative SEM images (**a**,**b**) and TEM images (**c**,**d**) of the Ni_3_Si_2_O_5_(OH)_4_ catalyst. Reproduced with permission from ref [92]. Copyright (2019), Elsevier Inc., Amsterdam, The Netherlands.

Liu et al. were interested in using non-precious metal for the preparation of heterogeneous catalysts with high catalytic activity for the selective oxidation of styrene. Hierarchical hollow nickel silicate (Ni_3_Si_2_O_5_(OH)_4_) microflowers assembled from a uniform and numerous well-defined Ni_3_Si_2_O_5_(OH)_4_ nanosheets were prepared by the authors using a one-pot hydrothermal method [92]. The interesting structure of the hollow Ni_3_Si_2_O_5_(OH)_4_ microflowers exhibited a high surface area of 177 m^2^g^−1^ and an average pore size of 3.9 nm (Figure 19).

**Scheme 14:**
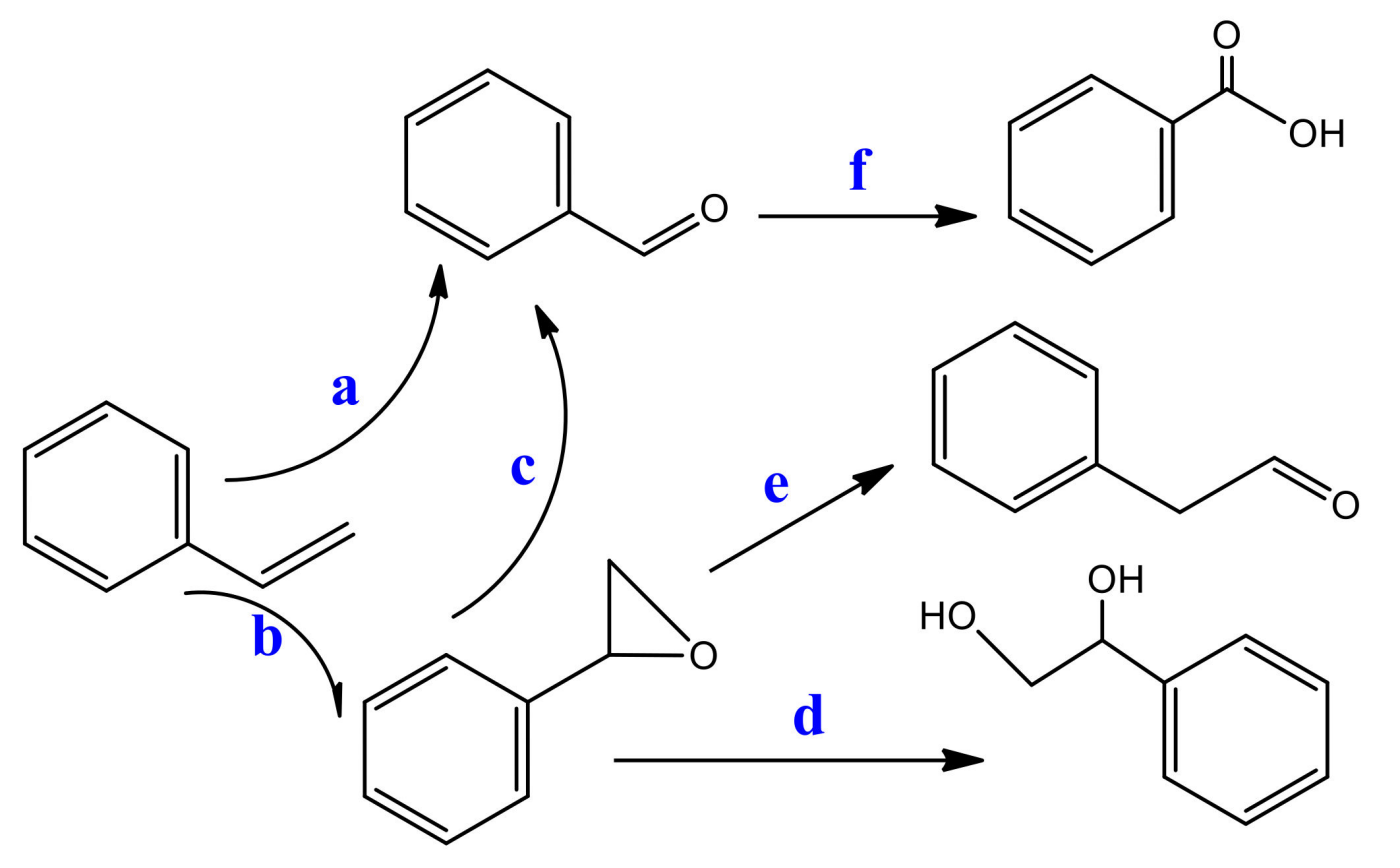
Proposed reaction pathway for the styrene oxidation reaction. Reproduced with permission from ref [92]. Copyright (2019), Elsevier Inc., Amsterdam, The Netherlands.

The catalytic application of this material for the selective oxidation of styrene has disclosed an attractive performance, reaching a remarkable styrene conversion of 99% and a high selectivity of 81% to benzaldehyde, the main product obtained, when H_2_O_2_ was used as the oxidant. Although a higher selectivity of benzaldehyde was achieved for the Ni/SiO_2_ catalysts, a much lower catalytic activity was observed than that of the Ni_3_Si_2_O_5_(OH)_4_. The recycling performance of the catalyst was also assessed, allowing the reuse five times without a significant decrease in conversion and selectivity. The spent catalyst showed no apparent change compared to that of the fresh catalyst. The good catalytic activity of the hollow Ni_3_Si_2_O_5_(OH)_4_ microflowers was attributed to its hierarchical mesoporous architecture, offering adequate active sites, as Ni(II) species, for the catalytic reaction, but also promoting the mass transport of molecules within the channels. The authors postulated that the interaction of Ni(II) species with hydrogen peroxide to form Ni(III)-peroxide species that can react with the styrene molecules (Scheme 14). The styrene oxide formed can further undergo C=C bond cleavage due to the nucleophilic attack of H_2_O_2_ to produce benzaldehyde as the main product. Styrene oxide can be additionally converted into 1-phenyl-1,2-ethanediol (pathway d) and phenylacetaldehyde (pathway e), while benzaldehyde could be further oxidized into benzoic acid (pathway f). The styrene that was initially produced oxide was mainly converted into benzaldehyde.

Jiang et al. explored the catalytic activity of Ag-Co-MCM-41 catalysts with different metal loadings, synthesized through a hydrothermal method, applied to selective styrene oxidation [93]. Characterization studies proved the successful introduction of Ag and Co into the mesoporous network of MCM-41 and confirmed the well-preserved structure of the mesoporous material. The liquid-phase catalytic oxidation of styrene over these catalysts was investigated using H_2_O_2_ as an oxidizing agent (Table 12).

The authors addressed the influence of metal loading, catalyst dose, temperature, reaction time and styrene/oxidant molar ratio on styrene conversion, yield and selectivity to benzaldehyde. Optimum reaction conditions are as follows: catalyst dose of 3 wt % of the reactants, temperature reaction 60 °C, 7 h, *n*_styrene_/n_H2O2_ of 1:3, and a metal load of 0.01. The catalytic performance of Ag-Co-MCM-41 catalyst towards the oxidation of styrene was enhanced when compared with that of Ag-MCM-41 and Co-MCM-41, most likely due to the co-introduction of silver and cobalt into MCM-41 mesoporous molecular sieves that could assist the catalysis together (Table 12). In addition, the reusability of the catalysts was evaluated, with the reuse of Ag-Co-MCM-41 catalyst five times without significant loss of activity, suggesting the robustness of the material.

In 2020, Andas and colleagues prepared Ni-MCM-41 and Co-MCM-41 upon the introduction of heteroatoms into the framework of a mesoporous MCM-41 synthesized from silica extracted from rice husk ash [94]. The successful substitution of Si^4+^ with Ni and Co cations was confirmed by the shifting in the siloxane (Si-O-Si) bond as well as the occurrence of nickel phyllosilicates from the FTIR and DR/UV-vis analysis. The mesoporous behavior was unchanged upon the incorporation of the metal species into the MCM-41 framework. MCM-41 silica and the modified MCM-41 catalysts were evaluated in the liquid-phase oxidation of styrene for 4 h, with varying reaction parameters, as temperature, catalyst loading, solvent and molar ratio of styrene: H_2_O_2_. Under the optimum condition, the activity of the catalysts was found to increase according to the trend: Ni-MCM-41 (80%) > Co-MCM-41(57%) > MCM-41 (41%). Benzaldehyde was the only product detected over Ni-MCM-41 and Co-MCM-41, while for MCM-41, benzaldehyde (96%) and styrene oxide (4%) were observed (Table 13).

According to the authors, the surface characteristics and acidity strength of the materials were the factors that govern the catalytic process. The detection of styrene oxide indicated a free-radical mechanism is prevalent for styrene oxidation under the studied reaction condition. Negligible leaching to the solution medium was observed, and outstanding activity during successive catalytic runs validates the heterogeneous nature of Ni-MCM-41. Additionally, this study exemplifies a successful conversion of biomass wastes into value-added products.

## 6. Other Materials

In this section, we include relevant contributions on heterogeneous catalysts for the selective oxidation of styrene to benzaldehyde that make use of support materials distinct from those categorized in the above sections.

In 2012, Islam and colleagues reported the use of a polymer-anchored Cu(II) catalyst for the oxidation of olefins and alcohols with TBHP [7]. The catalyst oxidized styrene to benzaldehyde, obtaining good-to-moderate yields up to 97% selectivity to benzaldehyde at 90% styrene conversion. The catalyst was easily recovered and recycled without losing its catalytic performance.

**Figure 20:**
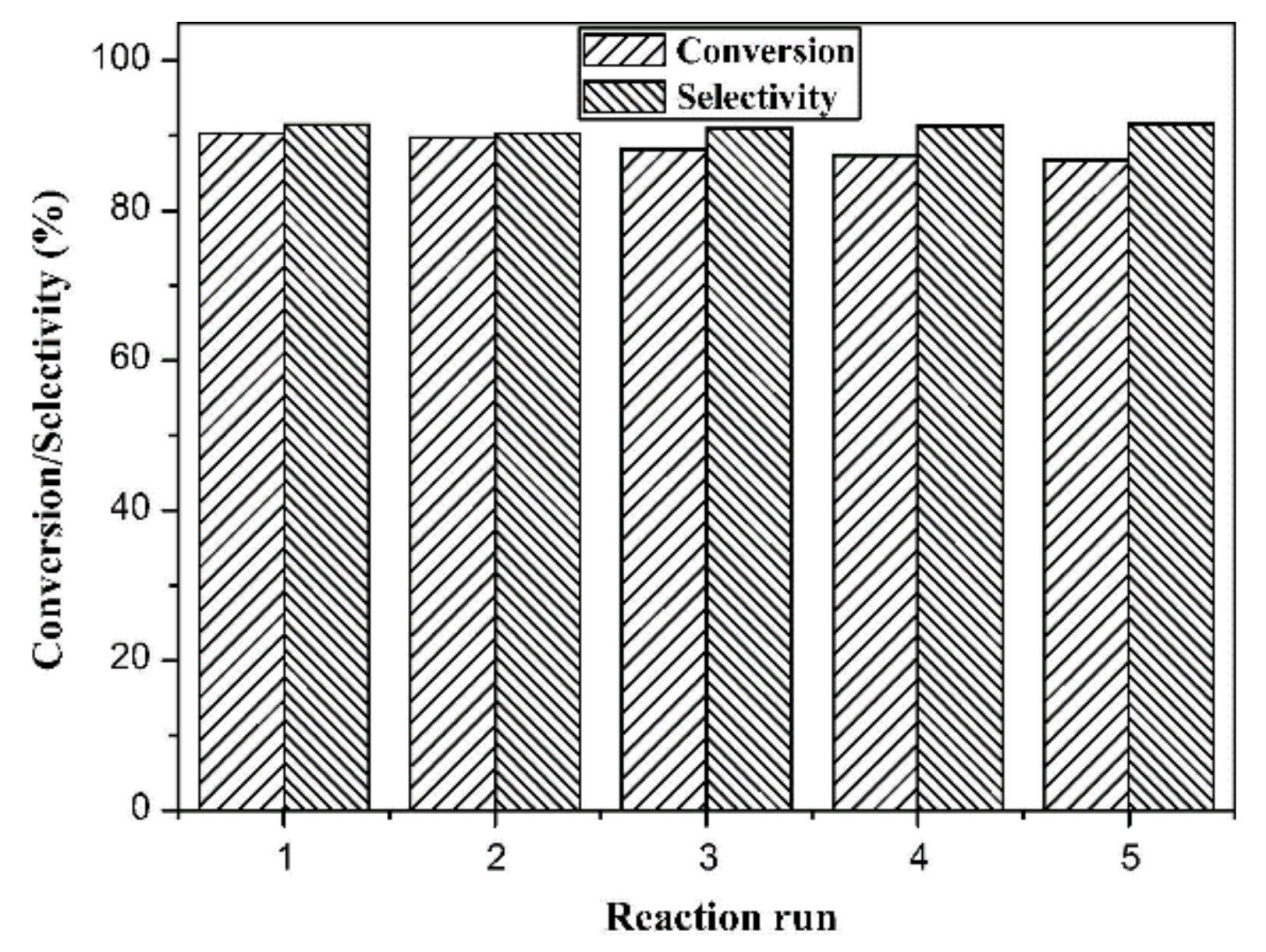
Reuse of the catalyst Ce_0_._3_Co_0_._7_Fe_2_O_4_. Reaction conditions: styrene: 2.0 mL (17.4 mmol), catalyst: 15.0 mg (0.06 mmol), styrene/H_2_O_2_:2:3, 1,4-dioxane: 10 mL, 90 °C, 9 h. Reproduced with permission from ref [95]. Copyright (2016), Elsevier Inc., Amsterdam, The Netherlands.

The selective oxidation of styrene catalyzed by cerium-doped cobalt ferrite nanocrystals was studied by Tong and colleagues in 2016 [95]. Ce-doped cobalt ferrite materials, Ce_x_Co_1x_Fe_2_O_4_ (x = 0.1, 0.3, 0.5), were obtained by a sol–gel auto-combustion route and further assessed as catalysts for the oxidation of styrene using H_2_O_2_. The Ce-doped samples were more efficient as catalysts for the oxidation of styrene to benzaldehyde when compared with pristine CoFe_2_O_4_ and CeO_2_. The best performing catalyst was Ce_0_._3_Co_0_._7_Fe_2_O_4_, achieving 90% styrene conversion and 92% of benzaldehyde selectivity at 90 °C for a 9 h reaction. The catalyst was magnetically separated and successfully reused in five consecutive runs (Figure 20).

**Figure 21:**
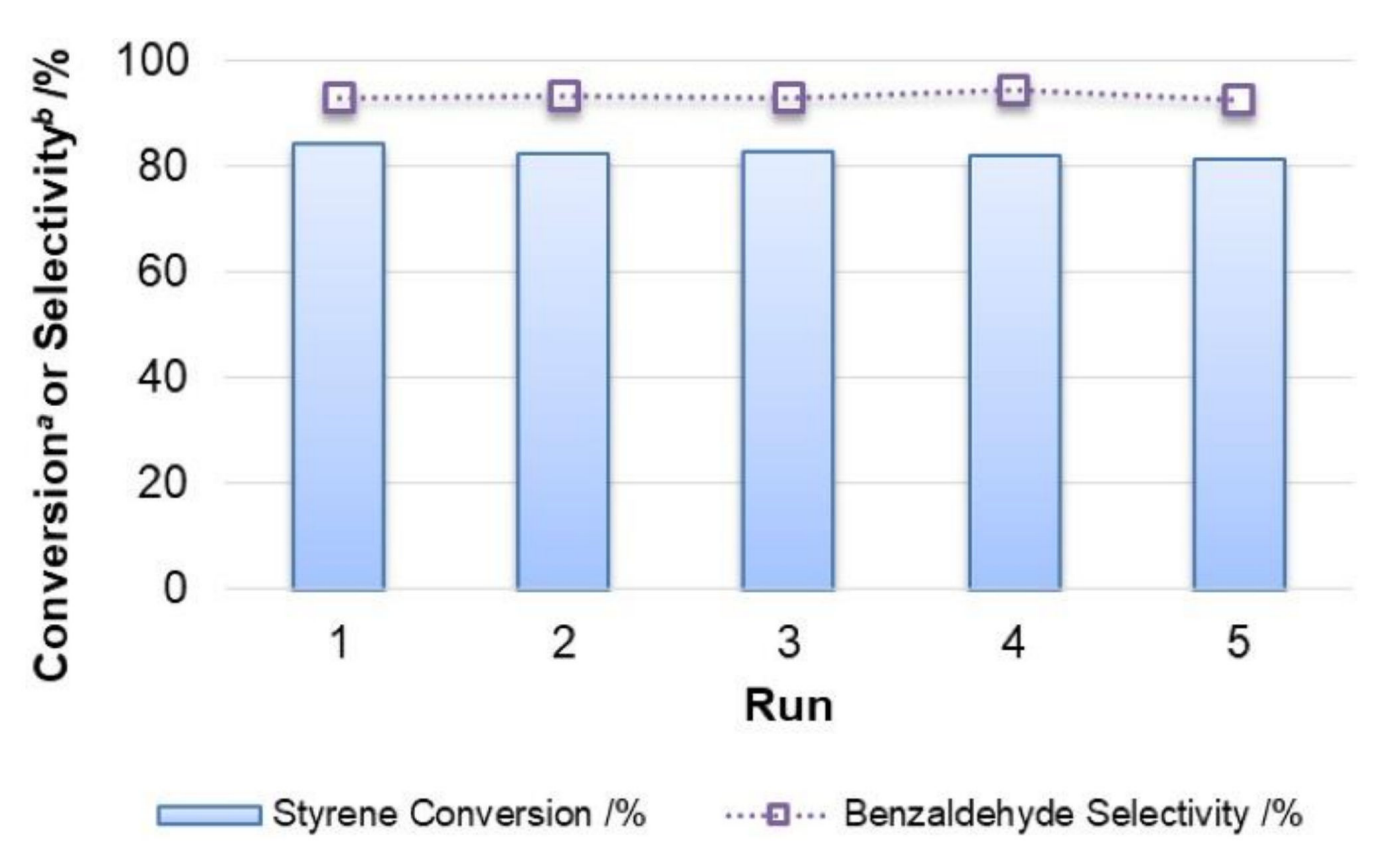
Catalyst recycling in the solvent-free MW-assisted oxidation of styrene catalyzed by MnFe_2_O_4_. Reaction conditions: MnFe_2_O_4_ (30 mg, 0.13 mmol), styrene (2.5 mmol), TBHP (2.5, 5.0, 7.5 or 10.0 mmol), T = 80 °C, t = 3 h. Molar conversion (%) based on styrene, i.e., moles of converted styrene per 100 mol of initial styrene. Molar selectivity (%) of product, i.e., moles of formed benzaldehyde or styrene oxide per 100 mol of converted styrene. Reproduced with permission from ref [96]. Copyright (2018), Elsevier Inc., Amsterdam, The Netherlands.

Martins and coworkers proposed a greener procedure for the catalytic oxidation of styrene over magnetic metal-transition ferrite nanoparticles [96]. An MW-assisted protocol was used to selectively obtain benzaldehyde from styrene using ferrite magnetic nanoparticles, MFe_2_O_4_ [M = Mn^2+^, Fe^2+^, Co^2+^, Ni^2+^, Cu^2+^ or Zn^2+^] as catalysts. The best catalytic performance towards the selective production of benzaldehyde (93% selectivity at 86% styrene conversion) was observed for the CoFe_2_O_4_ nanoparticles in 3 h at 80 °C using TBHP. The catalyst was readily isolated, and no loss of activity was observed upon reuse up to 5 consecutive runs (Figure 21). The low E-factor (mass ratio of waste to desired product) value (2.5) was pointed out by the authors as indicative of the ecofriendly nature of the proposed catalytic system.

**Figure 22:**
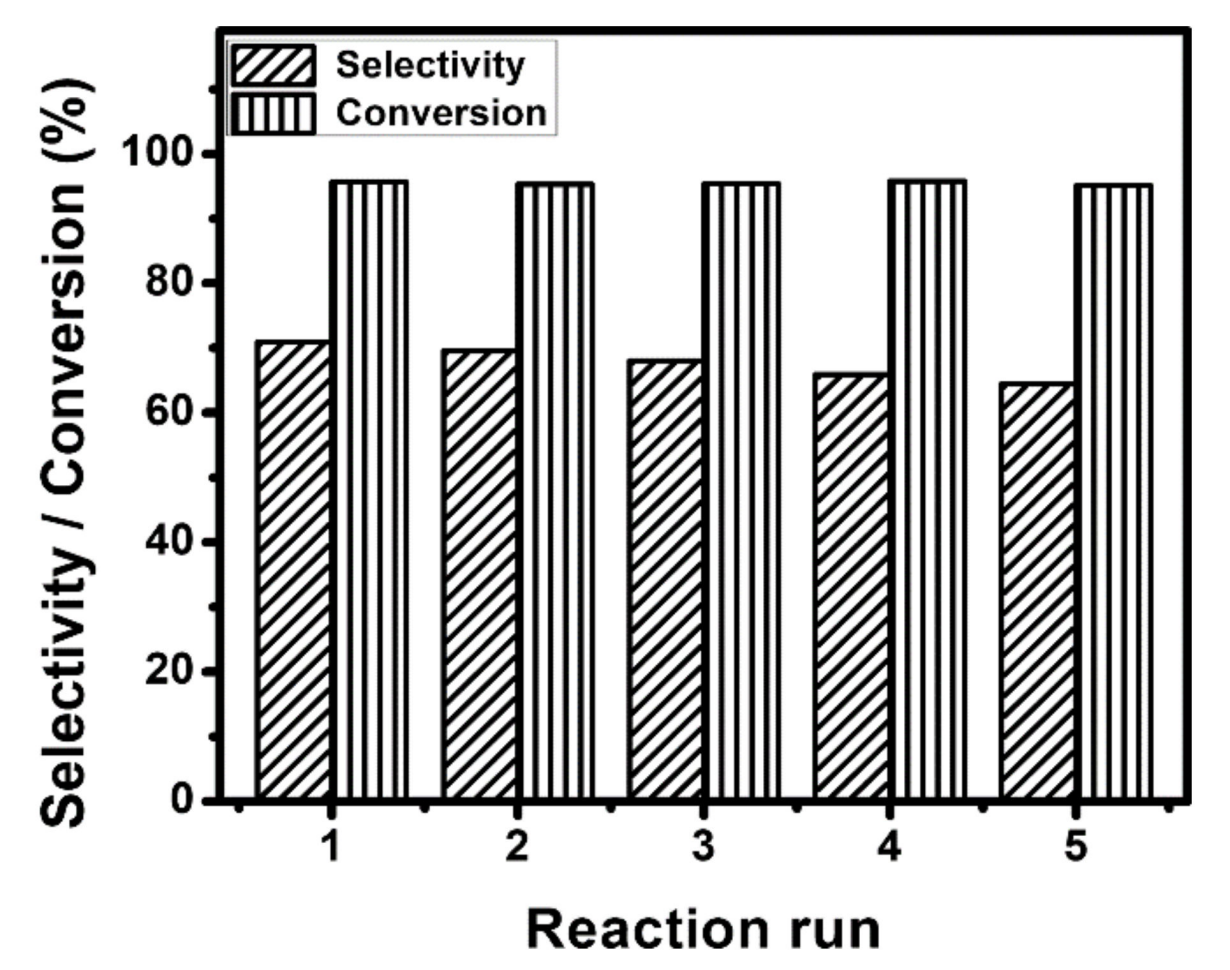
Recycling of the catalyst Ba_1_._8_Ce_0_._2_Co_2_Fe_12_O_22_ ferrite in styrene oxidation. Reproduced with permission from ref [97]. Copyright (2020), Springer Nature, Amsterdam, The Netherlands.

In 2020, Ce-doped Y-type barium hexaferrites Ba_2−x_Ce_x_Co_2_Fe_12_O_22_ (x = 0.0, 0.1, 0.2, and 0.3) were synthesized using the sol–gel auto combustion method and investigated as catalysts for the conversion of styrene to benzaldehyde [97]. The effects of catalyst amount, reaction temperature and time, solvent and oxidizing agent in the selective styrene oxidation to benzaldehyde were examined. Pure Y-type barium hexaferrite (BrM) samples showed total conversion of styrene and 66% isolated yield for benzaldehyde at 120 °C for 24 h in the presence of trifluoroacetic acid as an oxidant. The effect of Ce doping on the benzaldehyde selectivity was also analyzed at optimized conditions and found that cerium-doped samples showed excellent catalytic performance. The catalytic efficiency was significantly enhanced, and maximum catalytic activity was observed for Ba_1_._8_Ce_0_._2_Co_2_Fe_12_O_22_ ferrite, achieving 71% selectivity with the same reaction conditions. The catalyst was magnetically separable and used in five consecutive cycles, with no significant loss of catalytic activity (Figure 22).

Alkaline tantalates (LiTaO_3_, NaTaO_3_, KTaO_3_) prepared using the citrate method were evaluated as heterogeneous catalysts in the selective oxidation of styrene towards benzaldehyde by Leal Marchena et al. in 2018 [98]. The increased catalytic activity observed was correlated with the atomic radii of the alkaline cation. The highest styrene conversion (58%) and benzaldehyde selectivity (77%) were found for KTaO_3_ that can be attributed to the lower crystallinity, higher surface area and presence of carbonate potassium as segregated phases. This catalyst was recycled six times, with no significant loss of activity.

The oxidation of styrene using TBHP over zirconia supported mono-copper substituted phosphotungstate was studied by Sadasivan and coworkers [99]. The heterogeneous catalyst involving mono-copper substituted phosphotungstate and hydrous zirconia was prepared using the wet impregnation method and further evaluated for solvent-free oxidation of styrene. To understand the role of the support, the catalytic activity was compared with that of unfunctionalized PW_11_Cu. Additionally, the role of each component was clearly revealed by a thorough reaction kinetic study using both catalysts.

The selective oxidation of styrene to benzaldehyde by using a TiO_2_–H_2_O_2_ catalytic system was studied in 2019 by Ito et al. [100]. The oxidation promoted multistep reactions from styrenes, including the cleavage of a C=C double bond and the addition of an oxygen atom selectively and stepwise to deliver the corresponding benzaldehydes yields up to 72%. The reaction processes were spectroscopically demonstrated by fluorescent measurements under the presence of competitive scavengers. The absence of the signal from OH radicals suggests the participation of hydroperoxy radicals (**^•^**OOH) and superoxide radicals (**^•^**O_2_^−^) in the selective oxidation from styrene to benzaldehyde.

**Figure 23:**
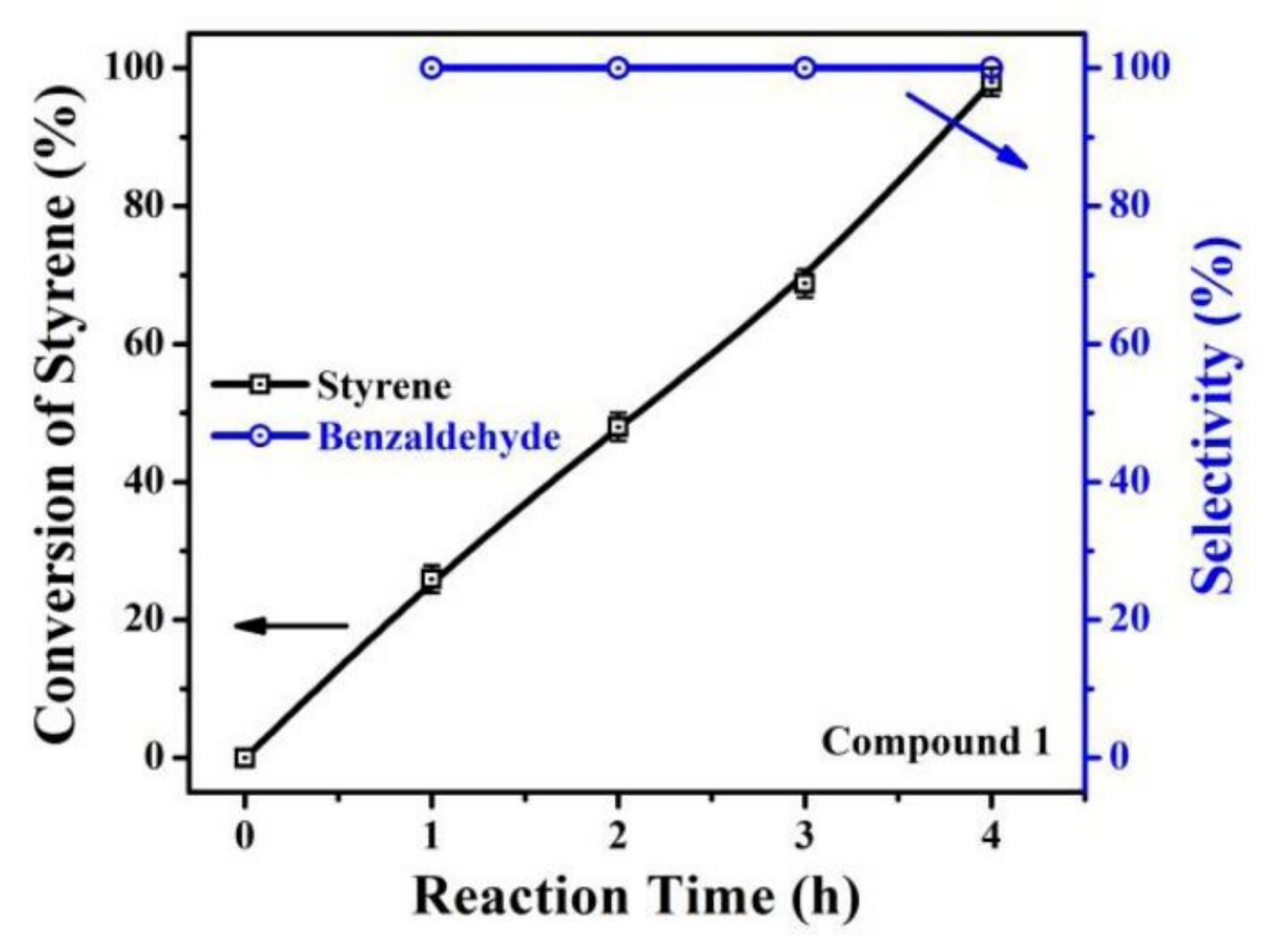
Conversion of styrene and selectivity of benzaldehyde in the selective oxidation of styrene over [Co(H_2_O)_3_]_2_(ttb)_2_ [HBW_12_O_40_].2H_2_O. Reproduced with permission from ref [101]. Copyright (2020), Informa UK Limited, trading as Taylor and Francis Group.

A Keggin-type POM-based supramolecular complex, [Co(H_2_O)_3_]_2_(ttb)_2_ [HBW_12_O_40_]_2_H_2_O) (ttb = 1,3,5-tri(1,2,4-triazol-1-ylmethyl)-2,4,6-trimethyl benzene) was synthesized via the hydrothermal method by Cui and coworkers and applied to selective styrene oxidation [101]. The [HBW_12_O_40_]_4_ polyoxoanions were immobilized between the layers through intermolecular interactions, producing a POM-encapsulated supramolecular network. [Co(H_2_O)_3_]_2_(ttb)_2_ [HBW_12_O_40_]_2_H_2_O) as catalyst displayed a synergistically improved performance for the oxidation of styrene, achieving 99% selectivity of benzaldehyde at 98% conversion of styrene in 4 h (Figure 23), that is about five times higher than the catalytic activities observed for the single components (Co(OAc)_24_H_2_O or K_5_BW_12_O_40_). Comparative analyses demonstrated that the high catalytic performance of the catalyst could be mainly ascribed to the synergistic effect between the unsaturated metal sites and the polyoxoanions.

Zhou and coworkers synthesized a new polyoxovanadate through a hydrothermal procedure and applied it to the selective oxidation of styrene [102]. The new bicadmium-substituted vanadosilicate cluster, [Cd(en)_2_]_2_[(en)_2_Cd_2_Si_8_V_12_O_40_(OH)_8_(H_2_O)_0_._5_]^4−^ (en = ethylenediamine) was derived from the classical {V_18_O_42_} cluster by replacing six {VO_5_} square pyramids with four {Si_2_O_7_} and two [Cd(en)]^2+^ groups. This compound exhibited a unique 1D chainlike structure that subsequently stacked into a 3D supramolecular framework. The prepared compound performed as an efficient heterogeneous catalyst towards the selective oxidation of styrene to benzaldehyde, reaching a conversion of 97% at 87% selectivity to benzaldehyde in 8 h.

Ag/AgBr/TiO_2_ ternary component nanotubes were used as heterogeneous photocatalysts towards the selective oxidative cleavage of C=C double bond of styrene to benzaldehyde under visible light at room temperature [103]. The reaction conditions, such as photocatalyst load, solvents, irradiation sources, and atmosphere, were investigated. Under the optimized reaction conditions, scaled up for the large-scale synthesis of benzaldehyde, 40% yield of benzaldehyde was observed after 4 h and 90% after 8 h. The method developed by the authors showed some advantages, for instance, with no requirement of oxidant, simple and short reaction time and easy separation of products. Furthermore, the Ag/AgBr/TiO_2_ nanotubes were reused several times without decreasing their photocatalytic activity. A wide variety of carbonyl compounds were also successfully synthesized through the developed photocatalytic process.

## 7. Summary and Outlook

This review offers an overview of the studies on the selective oxidation of styrene to benzaldehyde under heterogeneous catalyzed conditions from 2011 onward. The challenging oxidation process regarding the selective conversion of styrene to benzaldehyde is very significant in the synthesis of fine compounds and the production of commodity chemicals. The research community has a great interest in the pursuit of more efficient and greener methods for styrene oxidation; for that, a wide selection of catalysts have been explored under heterogeneous conditions, overcoming the drawbacks of homogeneous catalysts. The immobilization or entrapment of metal catalysts over different solid supports, such as carbon nanotubes, silica, metal oxides, metal–organic frameworks, polymers, etc., has provided an enhanced activity and selectivity towards benzaldehyde in styrene oxidation. The most employed metals for heterogenization on solid supports are Cu, Co, Cr and V, whether as ions, oxides, complexes or nanoparticles. The most-reported oxidants in these synthetic routes are TBHP and hydrogen peroxide. Among all the classes of supports reported in this review, one can find examples of heterogeneous catalysts that provide excellent selectivities to benzaldehyde for the oxidation of styrene. Nevertheless, in most of the cited cases, styrene conversion is obtained from moderate to good values, achieving high conversion in a limited number of studies. Comparison between the different classes of catalysts is not straightforward, given the limited number of examples in the literature for styrene oxidation and also considering the different reaction conditions reported by the authors. Synergistic effects between the metal and the support are pointed out in most of the included literature as responsible for the improved performance towards selective styrene oxidation demonstrated by the heterogeneous catalysts. Mechanistic considerations account for two reactional paths to produce benzaldehyde. The reaction proceeds either through a radical mechanism, in that the hydroperoxyl or hydroxyl radicals add to the C=C bond of styrene and further oxidative cleavage to form benzaldehyde is observed, or through a reaction of styrene with an oxidant molecule to form styrene oxide, further originating an intermediate upon nucleophilic attack by another molecule of oxidant, followed by carbon bond cleavage producing benzaldehyde. However, substantial efforts have been allocated to develop greener routes for this process, as, for example, using hydrothermal routes for the preparation of the catalysts and solvent-free conditions, a considerable amount of research on providing better performing catalysts for styrene oxidation is underway in the near future, aiming for stable and reusable catalysts, simple preparation procedure and mild reaction conditions.

## Figures and Tables

**Table 1:** Effect of the selected oxidant on styrene oxidation catalyzed by [Cu_2_(bipy)_2_(btec)]_∞_ [15].

Oxidant	Conversion (%) (TOF h^−1^)	Selectivity (%)	
		BZ	SO
TBHP ^(a)^	61(41)	26	32
TBHP ^(b)^	55(37)	32	37
O_2_^(a)^	0(0)	0	0
O_2_^(b)^	0(0)	0	0
H_2_O_2_^(c)^	37(25)	100	0

Reaction conditions: ^(a)^ solvent: 1,2-dichloroethane; time: 6 h; temperature: 75 °C. ^(b)^ Solvent: n-decane; time: 6 h; temperature: 75 °C. ^(c)^ Concentration of copper in the reaction medium is 62%. S/C ratio 400:1 (total moles of products)/(moles of catalyst × 6 h). TOF: turnover frequency; BZ: benzaldehyde; SO: styrene oxide.

**Table 2:** Oxidation of styrene over different catalysts ^(a)^ [18].

Catalyst	Conversion (%)	Selectivity BZ (%)
MOF-74 (Cu)	1	100
MOF-74(Co)	47	35
Cu(NO_3_)_2_	1	100
Co(NO_3_)_2_	51	16
MOF-74(Cu-90/Co-10)	10	75
MOF-74(Cu-75/Co-25)	17	50
MOF-74(Cu-30/Co-70)	30	43
91% MOF-74(Cu-30/Co-70) + 9% MOF-74(Co)	1	100
77% MOF-74(Cu-30/Co-70) + 23% MOF-74(Co)	4	85
32% MOF-74(Cu-30/Co-70) + 68% MOF-74(Co)	18	57
26% Cu(NO_3_)_2_ + 74% MOF-74(Co)	35	34
28% Cu(NO_3_)_2_ + 72% Co(NO_3_)_2_	45	18

^(a)^ Reaction conditions: 50 mg (ca. 0.15 mmol) of catalyst, 5 mL styrene (43.6 mmol), and O_2_ balloon at 80 °C for 20 h.

**Table 3:** Selective oxidation of styrene over selected metal–organic frameworks (MOFs) [22].

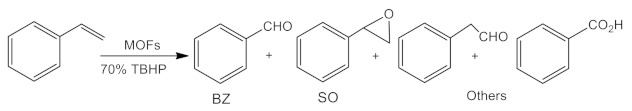				
Catalyst	Conversion (%)	Selectivity (%)		
		BZ	SO	Others
Mn-MIL-100	73	82	12	6
Fe-MIL-100	74	76	14	10
Fe-MIL-101	84	68	25	8
Cr-MIL-101	47	91	9	-

Reaction conditions: n_TBHP_:n_Styrene_ = 3:1; reaction temperature= 70 °C; time= 6 h; catalyst dosage = 5 wt %; solvent: CH_3_CN; BZ: benzaldehyde; SO: styrene oxide.

**Table 4:** Selective oxidation over different catalysts ^(a)^ [37].

Catalyst	Pd (mmol)	Conversion (%)	Selectivity (%)	
			BZ	SO
Y-bpdc	0	0	-	-
Y-bpydc_0_._8_/bpdc_0_._2_	0	0	-	-
Y-bpydc_0_._8_/bpdc_0_._2_	0	50	85	13
Pd(II)-Y-bpydc_0_._8_/bpdc_0_._2_	0.024	89	82	14
Pd(II)-Y-bpydc_0_._5_/bpdc_0_._5_	0.015	62	86	12
Pd(II)-Y-bpydc_0_._2_/bpdc_0_._8_	0.006	45	90	9
PdCl_2_	0.024	54	78	19
PdCl_2_	0.024	90	65	25

^(a)^ Reaction conditions: styrene (5 mmol), catalyst (0.005 mmol, 0.1 mol%), O_2_ (1 atm), 80 °C and 5 h.

**Table 5:** Styrene oxidation catalyzed by [VO(HNIMM)))(H_2_O)]-Y [45].

Catalyst	Conversion (%)	Selectivity (%)	
		BZ	SO
[VO(HNIMMPP)(H_2_O)]-Y	93	46	9
[Mn(HNIMMPP)(H_2_O)_3_]-Y	51	42	11
[Fe(HNIMMPP)(H_2_O)_3_]-Y	61	49	8
[Co(HNIMMPP)(H_2_O)]-Y	82	47	5
[Ni(HNIMMPP)(H_2_O)]-Y	90	51	1
[Cu(HNIMMPP)(H_2_O)]-Y	88	57	2

Reaction conditions: 30 mmol of styrene, 60 mmol of TBHP, 10 mL of acetonitrile, 0.07 g of catalyst, 80 °C, 10 h.

**Table 6:** Selective oxidation of styrene to benzaldehyde using a Cr-ZSM-5 zeolite catalyst and using acetonitrile as solvent.

Temp. (°C)	Time (h)	Conversion (%)	Selectivity (%)
50	4	56	76
	5	66	76
	6	74	76
60	4	64	75
	5	71	75
	6	79	75
70	4	66	75
	5	77	75
	6	86	75

**Table 7:** Optimization of reaction conditions for styrene oxidation to benzaldehyde catalyzed by 1@S0_Na [71].

Solvent	*n*_(H2O2)/_*n*_(catalyst)_ × 10^3^	Conversion (%)	Selectivity (%)
CH_3_CN	1.3	25	45
CH_3_CN	0.7	22	68
CH_3_CN	0.3	20	79
H_2_O	1.3	8	36
H_2_O	0.7	6	42
H_2_O	0.3	7	40
-	1.3	50	32
-	0.7	42	56
-	0.3	43	81

Reaction conditions: 1.5 mL of solvent, 1 mmol of styrene, 3 μmol of metal in hydrochar, 80 °C, 2 h.

**Table 8:** Recycling experiments for Cu-SBA-15 in styrene oxidation [84].

Cycle	Conversion (%)	Selectivity (%)		
		BZ	BA	SO
1	84	84	16	0
2	87	82	18	0
3	86	81	19	0

**Table 9:** Catalytic performance of vanadium-containing catalysts [86].

Catalyst	V/Si (%)	Conversion (%)	BZ Selectivity (%)
MCM-48	0.0	-	-
2V-MCM-48	0.7	22	95
4V-MCM-48	1.3	39	96
6V-MCM-48	2.0	44	95
8V-MCM-48	2.9	40	94
V-MCM-48	1.4	26	91
4V-MCM-48	1.2	10	92
8V-MCM-48	3.0	18	90
V_2_O5	-	11	91

Reaction conditions: temperature, 30 °C; styrene, 2, mL; H_2_O_2_, 2 mL; catalyst, 0.1 mg; reaction time, 12 h and acetonitrile, 20 mL.

**Table 10:** Recyclability test for styrene oxidation over 10CuNiSiO catalyst [89].

Cycle	Conversion (%)	Selectivity (%)		
		BZ	BA	Others
1	88	62	25	13
2	86	61	27	12
3	84	60	28	11

Reaction conditions: 0.96 mmol styrene in 2.5 mL, CH_3_CN, styrene: TBHP 1:15, reaction temperature 40 °C. BZ: benzaldehyde; BA: benzoic acid.

**Table 11:** The effect of various catalysts for the oxidation of styrene to benzaldehyde with H_2_O_2_ [91].

Catalyst	Yield%)	Conversion (%)	Selectivity (%)
SBA-15	-	-	-
PW_12_	7.1	7.7	92.4
PW_12_/SBA-15–8.9	6.1	6.1	100
PW_12_/SBA-15–14.5	22.6	22.6	100
PW_12_/SBA-15–15.7	22.9	22.9	100

Catalyst, 100 mg; acetonitrile, 5 mL; styrene, 1.0 mmol; H_2_O_2_, 4 mmol; 70 °C; 24 h.

**Table 12:** Catalytic oxidation of styrene over different catalysts [93].

Sample	Conversion (%)	Selectivity (%)	Yield (%)
0.01-Co-MCM-41	36	44	16
0.01-Ag-MCM-41	29	38	11
0.01-Ag-Co-MCM-41	45	53	24

Reaction conditions: catalyst dose: 3 wt % of the reactants; temperature: 60 °C; metal load: 0.01; time: 7 h; n_styrene_/n_H2O2_:1:3.

**Table 13:** Catalytic activities over the different catalyst and reuse cycles of Ni- Ni-MCM-41 in the oxidation of styrene [94].

Catalyst	Conversion (%)	Selectivity (%)	
		BZ	SO
No catalyst	0	-	-
MCM-41	41	96	4
Ni-MCM-41	80	100	0
Co-MCM-41	57	100	0
Ni-MCM-41 1st cycle	75	100	0
Ni-MCM-41 2nd cycle	74	100	0
Ni-MCM-41 3rd cycle	71	100	0

## Data Availability

No new data were created in this study.
